# G-quadruplex landscape and its regulation revealed by a new antibody capture method

**DOI:** 10.18632/oncotarget.28564

**Published:** 2024-03-14

**Authors:** Subhamoy Datta, Manthan Patel, Chakkarai Sathyaseelan, Chandrama Ghosh, Akanksha Mudgal, Divyesh Patel, Thenmalarchelvi Rathinavelan, Umashankar Singh

**Affiliations:** ^1^HoMeCell Lab, Discipline of Biological Engineering, Indian Institute of Technology Gandhinagar, Gandhinagar, Gujarat 382355, India; ^2^Centre for Genomics and Child Health, Blizard Institute, Barts and The London School of Medicine and Dentistry, Queen Mary University of London, London E1 2AT, UK; ^3^Department of Biotechnology, Indian Institute of Technology Hyderabad, Kandi Campus, Telangana 502285, India; ^4^Azrieli Faculty of Medicine, Bar-Ilan University, Henrietta Szold 8A, Safed 1311502, Israel; ^5^Department of Biopharmacy, Medical University of Lublin, Lublin 20059, Poland; ^6^Research Programs Unit, Applied Tumor Genomics Program, Faculty of Medicine, University of Helsinki, Biomedicum, Helsinki 00290, Finland

**Keywords:** DNA G-quadruplexes, G4-ChIP, CGGBP1, CTCF, G/C-skew

## Abstract

Our understanding of DNA G-quadruplexes (G4s) from *in vitro* studies has been complemented by genome-wide G4 landscapes from cultured cells. Conventionally, the formation of G4s is accepted to depend on G-repeats such that they form tetrads. However, genome-wide G4s characterized through high-throughput sequencing suggest that these structures form at a large number of regions with no such canonical G4-forming signatures. Many G4-binding proteins have been described with no evidence for any protein that binds to and stabilizes G4s. It remains unknown what fraction of G4s formed in human cells are protein-bound. The G4-chromatin immunoprecipitation (G4-ChIP) method hitherto employed to describe G4 landscapes preferentially reports G4s that get crosslinked to proteins in their proximity. Our current understanding of the G4 landscape is biased against representation of G4s which escape crosslinking as they are not stabilized by protein-binding and presumably transient. We report a protocol that captures G4s from the cells efficiently without any bias as well as eliminates the detection of G4s formed artifactually on crosslinked sheared chromatin post-fixation. We discover that G4s form sparingly at SINEs. An application of this method shows that depletion of a repeat-binding protein CGGBP1 enhances net G4 capture at CGGBP1-dependent CTCF-binding sites and regions of sharp interstrand G/C-skew transitions. Thus, we present an improved method for G4 landscape determination and by applying it we show that sequence property-specific constraints of the nuclear environment mitigate G4 formation.

## INTRODUCTION

The containment and maintenance of the genomic DNA inside the human cellular nuclei is a tightly regulated process. Higher-order chromatin conformation and chromosomal territories exemplify the manifestation of an orderly containment of the genomic DNA at a broad scale [1, 2]. These arrangements selectively allow regulatory interactions between genomic regions separated by long distances or located *in trans*. It is well established that this orderly arrangement of the genome depends on interactions between DNA and chromatin regulatory proteins, such as CTCF [3] and its co-operators, such as CGGBP1 [4–6]. The complexity of such regulatory proteins and their cognate binding sites in the genome has increased in the course of evolution. Essentially, a significant amount of cellular resources are directed to ensure that the DNA does not randomly adopt conformations which would interfere with DNA-protein interactions [7, 8].

The DNA in the nucleus is under a state of flux through various processes that involve strand separation and new strand synthesis. In addition, certain functional regions of the genome, such as transcription enhancers and promoter elements, interact facultatively with transcription factors and may evade dense packaging with histone and related proteins in a cell-type and developmental state-specific manner. The DNA in these regions, often associated with G/C richness [9, 10], becomes amenable to adopt secondary structures. One such secondary structure of DNA that has attracted wide interest is the G4s [8]. The G4s form due to a preference for Hoogsteen base pairing over Watson-Crick base pairing in certain sequence contexts and involves four strands of DNA. The G4s could form from multiple parallel or antiparallel conformations of the same or different DNA strands. The variety of G4s discovered so far indicates that the structure formed is the lowest energy state conformation possible in a certain biochemical context, a process solely dependent on the chemical properties of the DNA, its nucleotide sequence, strandedness and the biochemical environment [11–13]. Much of our understanding of the nature of G4s comes from *in vitro* characterization of DNA secondary structures [14, 15]. A commonly accepted signature sequence for G4 formation is a tandem repeat of (G_3_-N_(1-7)_) four times on a single strand. However, in a complex mix of DNA, such as the human genome, single units of (G_3_-N_(1-7)_) from different molecules could collaborate and form intermolecular G4s [14, 15]. It is obvious that the formation of secondary structures such as G4s in cellular nuclei would be enhanced if the DNA escapes the constraints of the nuclear environment. Determination of the G4 landscape of the human genome has the potential to elucidate their biological significance.

The adoption of secondary structures such as G4s by the DNA is a hindrance to genomic DNA usage and maintenance. Hairpin loops and G4s formed by tandem repeats cause polymerase stalling and replication errors [16]. Repetitive sequences prone to the formation of quadruplex structures depend on helicases [17] for efficient replication and repair. These energy-dependent secondary structure resolution mechanisms collaborate with the constraints of the nuclear environment to mitigate secondary structure formation. More recently, regulatory functions have been attributed to quadruplex structures [8, 18–20]. Studies indicate that the regulation of expression of some genes depends on quadruplex formation in their *cis*-regulatory regions [21–23]. It remains unclear if the G4s formation in *cis*-regulatory regions is a consequence of nucleosome depletion and relaxation of constraints of the nuclear environment or if there are dedicated mechanisms to ensure the formation and stabilization of such regulatory quadruplexes. An increasing number of studies have reported an array of G4-binding proteins [24–28]. However, most of these studies do not distinguish between proteins binding to sequences capable of forming G4s or actual G4 structures. The consequences of protein-G4 interactions remain unknown for many such candidate quadruplex binding proteins. There is evidence that many helicases with RRM domains or RGG/UP1 motifs bind to and resolve the G4s [29–31]. Hundreds of other proteins have been identified as associated with G4s with no indications of any common quadruplex-specific binding domain [24].

While there is overwhelming evidence that G4s affect DNA-protein interactions, thereby affecting the epigenome, there is no evidence for a targeted G4 stabilizing mechanism in human cells. Most of these G4-binding proteins seem to have an affinity for generally GC-rich DNA and nucleosome-depleted regions, suggesting that their binding could exert constraints of the nuclear environment on G4 formation. The G4 landscape determination with knockdown of these G4-binders (and putative G4 regulators) will elucidate the role of these proteins in the quadruplex establishment in cells. A comprehensive determination of the G4 landscape and their regulation by quadruplex-binding proteins shall address the non-canonical G4s as well.

Valuable studies have described the genome-wide G4 landscapes in human cells [11, 32, 33]. The canonical (G_3_-N_(1-7)_)xn signature is only mildly enriched in these quadruplex pull-down-sequencing assays. Intriguingly, there is an unexplained bias for G/C-rich open chromatin and nucleosome-depleted regions in G4 landscape datasets, and the same G/C-rich regions are predominantly the binding sites for the G4-binding proteins [24, 32]. The influence of protein-binding on the capture and detection of G4s in these assays remains unknown. Interestingly, these studies also raise questions about the nature and scope of G4s at locations other than the nucleosome-depleted regions with low or no occupancy of G4-binding proteins.

Our current understanding of the DNA sequence properties associated with G4 formation *in cellulo* depends on the global G4 profiles, primarily generated using the G4-ChIP protocol [33]. Formaldehyde crosslinking, as used in conventional chromatin immunoprecipitation protocols, has been adopted in G4-ChIP as well. Although several G4-binding proteins have been described, no known dedicated G4-binding domains can stabilize the quadruplexes common to them. Formaldehyde crosslinking can efficiently capture only those G4s which are stably bound to proteins. This creates a detection bias against the G4s not bound to proteins. That formaldehyde is a passive DNA denaturing agent [34, 35] entails that protein unbound G4s would be underrepresented in the G4-ChIP. In addition, tetrads formed by non-G nucleotides also exist interspersed between G tetrads, with some even stabilizing the G4s to varying degrees [36–39]. Currently, we do not know if the large fraction of DNA pulled down by antibodies specific for G4s that does not show any (G_3_-N_(1-7)_)xn signature is an artifact or not. It remains unknown if the G/C-rich and non-G/C-rich G4s are regulated by the same or distinct mechanisms. Nevertheless, it remains established that although G/C richness is not an essential feature of quadruplex-forming regions, the G/C-rich sequences readily form stable quadruplexes *in vitro* and, to a certain extent, *in cellulo*. On the other hand, an overrepresentation of the (G_3_-N_(1-7)_)xn sequences in the G4-ChIP-seq could be due to the rapid adoption of quadruplex structures by (G_3_-N_(1-7)_)xn sequences post-fixation. DNA melted passively by formaldehyde as well as sheared by sonication into single-stranded overhangs would be amenable to forming quadruplexes post-fixation in the lysates or during any step before the anti-G4 antibody is added. The antibodies would capture the *in vitro* formed quadruplexes, which would be falsely represented in the G4-ChIP-seq results as bona fide quadruplexes *in cellulo*. Such a potential misrepresentation of the G4 landscape in the conventional G4-ChIP-seq experiments contaminates our understanding of *in cellulo* G4 landscape with *in vitro* formed quadruplexes.

Native G4 landscape has been described using CUT&Tag approaches with significant applications for single-cell mapping of G4s [40–43]. However, the Tn5 enzyme used in these essays show non-specific DNA-binding [43] and the elimination of false positives depends strongly on efficient washing away of pA-Tn5 conjugates. It is unclear how chromatin accessibility affects tagmentation and capture of regions using Tn5 [44, 45]. Most importantly, G4 formation is a feature of a single DNA strand at any genomic location. Although Tn5 can digest and tag single-stranded as well as double-stranded DNA, the tagging of single-stranded DNA takes place at only one of the two ends thereby rendering them not amplifiable in subsequent PCR steps [46, 47]. These CUT&Tag approaches do not address this feature of Tn5 on single-stranded DNA. One kind of G4s that could escape Tn5 tagmentation due to single strandedness of flanking DNA are those formed at regions with an interstrand G/C-skew.

Thus, multiple interrelated challenges affect our interpretation of the G4 landscape and its regulation. Does formaldehyde fixation truly capture the *in cellulo* G4s only? Do post-fixation formed quadruplexes *in vitro* get misrepresented as *in cellulo* G4s? Does G4 formation/stabilization depend on some regulatory protein(s)? Are there constraints of the nuclear environment exerted by G4-binding proteins on quadruplex formation? In this work, we shed light on these questions by using a new protocol which overcomes the limitations of existing protocols mentioned above. We have developed a ChIP-sequencing protocol for G4s to stabilize *in cellulo* quadruplexes and minimize any quadruplexes formed post-fixation *in vitro*. We use this protocol with a control DNA, having the (G_3_-N_(1-7)_)xn sequence signature, to establish the extent of constraints of the nuclear environment on quadruplex formation by (G_3_-N_(1-7)_)xn sequences *in cellulo* quantitatively. We use this protocol to describe the most native *in cellulo* G4 landscape and demonstrate that G4s form abundantly at satellite repeats while short-interspersed nuclear elements (SINEs) resist G4 formation.

CGGBP1 is a nuclear protein with a binding preference for G/C-rich repetitive DNA and interspersed repeats, including Alu-SINEs [48–50]. CGGBP1 regulates genomic stability under stress [51], cytosine methylation at DNA sequences with interstrand G/C-skew [50], an implied feature of the (G_3_-N_(1-7)_)xn signature attributed to G4 formation. We apply our protocol in cells depleted for CGGBP1 and find that CGGBP1 exerts constraints of the nuclear environment on G4 flux selectively. While CGGBP1 depletion leaves the low G4 prevalence at SINEs unchanged, it causes a net enhancement of G4 formation at regions with G/C-skew, which correlates with enhanced occupancy of CTCF at CGGBP1-regulated CTCF-binding sites. Our results suggest that the genome is under a constant widespread G4-flux, and the prevalence of G4 is determined by an interplay between the local sequence properties and their protein-binding. The new G4 capture method we present thus reveals a more widespread nature of the G4 landscape and its regulation.

## RESULTS

### G4s captured by G4-ChIP also form spontaneously on genomic DNA *in vitro*

The data derived from G4-ChIP are expected to represent an *in cellulo* G4 landscape. These *in cellulo* G4 landscapes are expected to be different from the G4 profile of purified genomic DNA where there are no constraints of the nuclear environment. We needed to compare the G4 landscapes derived from G4-ChIP with those derived entirely from purified genomic DNA *in vitro*. So we first performed *in vitro* G4 DNA immunoprecipitations (DNA-IPs) on purified genomic DNA from HEK293T in two different ways using the 1H6 antibody. In one of the DNA-IPs, called G4 DNA-IP^*nat*^, the double-stranded genomic DNA stored under nuturing conditions for seven days was incubated with the antibody. In a second DNA-IP, called (G4 DNA-IP^*denat*^), the same DNA was heat-denatured and snap-chilled just before the antibody incubation step. The quadruplexes expected to be pulled down in G4 DNA-IP^*nat*^ were stable G4s that would form spontaneously in largely double-stranded genomic DNA. In contrast, the G4s expected in G4 DNA-IP^*denat*^ would be those which form rapidly only when the DNA is rendered single-stranded (after heat denaturation and snap-chilling). In both cases, the binding of antibodies would stabilize the G4s for capture and characterization through sequencing. Both the DNA-IPs and their common input were sequenced (Supplementary Table 1) and compared.

**Figure 1:**
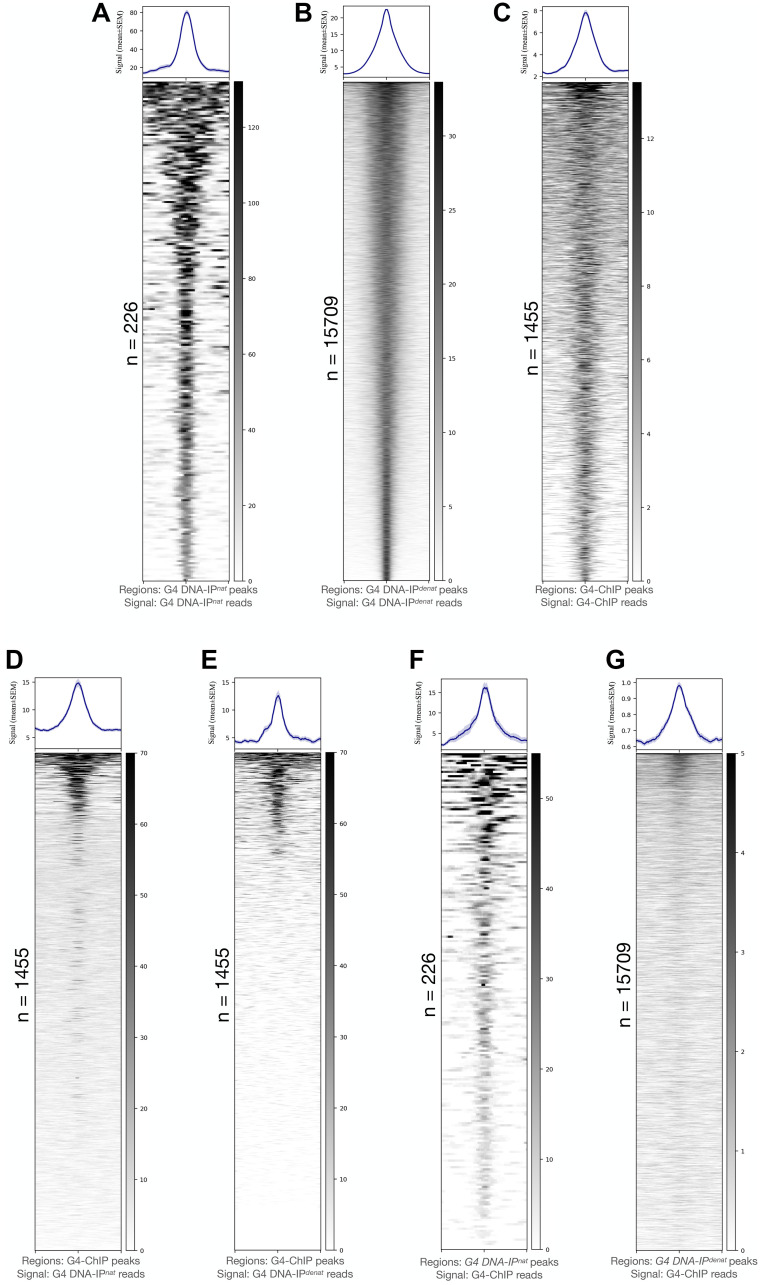
G4 potential of human genome characterized through G4 immunoprecipitation *in vitro* and its comparison with G4-ChIP output. (**A**–**C**) Peaks from G4 DNA-IP^*nat*^ (A), G4 DNA-IP^*denat*^ (B) and G4-ChIP (C) show specificities of G4 DNA pull-down. The G4 pull-down pattern gives rise to far fewer peaks in G4 DNA-IP^*nat*^ (A) on one extreme and a large number of peaks in G4 DNA-IP^*denat*^ (B) on the other. G4-ChIP peak count is higher but with a weaker mean signal than either G4 DNA-IP^*nat*^ or G4 DNA-IP^*denat*^. (**D**, **E**) A plotting on G4-ChIP peaks of signals from G4 DNA-IP^*nat*^ (D) or G4 DNA-IP^*denat*^ (**F**) shows that visibly more G4-ChIP peaks contain G4 DNA-IP^*nat*^ signals than G4 DNA-IP^*denat*^ signals. F and G: Most G4 DNA-IP^*nat*^ peaks (F) contained a strong G4-ChIP signal, whereas, on G4 DNA-IP^*denat*^ peaks (**G**), the G4-ChIP strong signals were restricted to a very small fraction of the peaks. The peak counts are mentioned beside the heatmaps. Signals are plotted as mean ± SEM on the Y-axes, and bed coordinates are arranged in decreasing order of the signal magnitude. All signals are plotted in regions 5 kb ± peak centers.

Compared to G4 DNA-IP^*nat*^, the G4 DNA-IP^*denat*^ showed a higher correlation with the input, suggesting that when the constraints of double-strandedness are overcome, G4 formation on the genomic DNA is rapid and widespread genome-wide *in vitro* (Supplementary Figure 1). This inference was supported by an analysis of how the G4 enrichments in G4 DNA-IP^*denat*^ and G4 DNA-IP^*nat*^ compared with each other. Using the same conditions of peak-calling, while 15709 peaks were discovered in G4 DNA-IP^*denat*^, only 226 peaks could be called in G4 DNA-IP^*nat*^ (Figure 1A–1B and Supplementary Table 2). The genome-wide signals from the two DNA-IPs were plotted reciprocally in their peaks (Supplementary Figure 2). Only a small fraction of the 15709 G4 DNA-IP^*denat*^ peaks were rich in signals from G4 DNA-IP^*nat*^ (Supplementary Figure 2A). On the contrary, nearly all of the 226 G4 DNA-IP^*nat*^ peaks were rich in G4 DNA-IP^*denat*^ signals (Supplementary Figure 2B). Strikingly, 92% of the G4 DNA-IP^*nat*^ peaks overlapped with just 1% of the G4 DNA-IP^*denat*^ peaks (Supplementary Figure 2C). An overwhelming 99% of the G4 DNA-IP^*denat*^ peaks found no overlap with the G4 DNA-IP^*nat*^ peaks (Supplementary Figure 2D). This suggested that a small fraction of the rapidly forming G4s in G4 DNA-IP^*denat*^ are stable quadruplexes which are also present in G4 DNA-IP^*nat*^. These results also showed that the immense G4-forming potential of human genomic DNA is restrained by its double-strandedness at most of the regions. At the same time, at some regions, the G4s prevail over the double-strandedness and form spontaneously.

Stable G4s form at the canonical (G_3_-N_(1-7)_)xn signature sequences. Also, it is known that the 1H6 antibody can capture secondary DNA structures without restricting to the canonical (G_3_-N_(1-7)_)xn signature sequences, especially upon denaturation [39]. Quality-filtered reads from the two assays were searched for the presence of G4-forming (G_3_-N_(1-7)_)xn sequences using *allquads* [15]. We observed that the (G_3_-N_(1-7)_)xn sequences were enriched in the G4 DNA-IP^*nat*^ sample, whereas the G4 DNA-IP^*denat*^ sample showed such sequences only at an expected level as in the input (Supplementary Table 3). The spontaneity of the formation of G4s and their stability have been shown to depend on the GC-content [52]. As expected, we observed that the GGG/CCC contents of G4 DNA-IP^*nat*^ peaks were significantly much lower compared to G4 DNA-IP^*denat*^ (Supplementary Figure 3). Thus, a majority of the G4s identified in G4 DNA-IP^*denat*^ seemed to be rapidly forming at G/C-rich DNA, where the energetic constraints of double-stranded DNA are overcome by heat denaturation. The structural turnover of the chromatin in a cell would allow abundant possibilities for such G4s to form.

We next asked how these two types of G4s are captured by the conventional G4-ChIP [33]. G4-ChIP was performed in HEK293T cells using a previously described protocol [33] (except that we used the 1H6 antibody) and compared its peaks (Figure 1C) and genome-wide signal with those of G4 DNA-IP^*denat*^ and G4 DNA-IP^*nat*^. Although the signals from the three experiments were highly uncorrelated with each other, the G4-ChIP and G4 DNA-IP^*nat*^ signals clustered together differently from G4 DNA-IP^*denat*^ (Supplementary Figure 4A). This weak similarity between G4-ChIP and G4 DNA-IP^*nat*^ was relevant as the 1455 G4-ChIP peaks contained strong signals mostly from G4 DNA-IP^*nat*^ (Figure 1D) and only weakly from G4 DNA-IP^*denat*^ (Figure 1E and Supplementary Figure 4B). The G4-ChIP peaks overlapped with over 70% of the G4 DNA-IP^*nat*^ peaks and less than 20% of the G4 DNA-IP^*denat*^ peaks. Reciprocally, the G4 DNA-IP^*nat*^ peaks contained a stronger and higher abundance of G4-ChIP signals (Figure 1F) as opposed to the G4 DNA-IP^*denat*^ peaks (Figure 1G). As expected, an allquads analysis showed that the G4-ChIP sample was also rich in (G_3_-N_(1-7)_)xn sequences compared to the respective input (Supplementary Table 4). A similar enrichment of (G_3_-N_(1-7)_)xn sequences was found in independently reported G4-ChIP data using BG4 antibody in HaCaT cells and normal human epidermal keratinocytes (NHEK) (Supplementary Table 5). Recapitulating the similarities between G4-ChIP peaks and G4 DNA-IP^*nat*^ peaks, the two peak sets also showed a similar abundance of relatively GGG/CCC-poor sequences (Supplementary Figure 3).

We could conclude from these findings that some regions of the genome, especially those with the (G_3_-N_(1-7)_)xn signature sequences and low GGG/CCC levels, are captured similarly *in vitro* (G4 DNA-IP^*nat*^) and from the chromatin using the G4-ChIP protocol. Although the G4-ChIP predominantly captured these spontaneously-forming G4s, it had a much lower representation of the G4s formed at GGG/CCC-rich regions and sequences poor in (G_3_-N_(1-7)_)xn signature. Although we found the latter type of G4s as the most prevalent in G4 DNA-IP^*denat*^, the reasons for their paucity in the G4-ChIP data, and the possibility of their existence and relevance in the chromatin, remained unaddressed by using the G4-ChIP protocol.

To investigate this further, we analyzed how various steps of the G4-ChIP protocol could affect the capture and enrichment of G4s. G4-ChIP relies on a protocol originally invented to capture DNA-protein interactions. In this protocol, the capture of G4s is expected to be favored by their stability, crosslinking to a protein by formaldehyde and no steric hindrance to antibody-G4 binding in the downstream steps. There is no evidence for G4 stabilization by G4-binding proteins. The similarity between the G4 profiles of G4 DNA-IP^*nat*^ (derived from protein-unbound DNA) and G4-ChIP suggests that such spontaneously forming G4s could be captured similarly from the DNA or chromatin after fixation and shearing by sonication, as the protocols of G4 DNA-IP^*nat*^, as well as of G4-ChIP, allow stabilization of G4 by their binding to the antibody during the incubation steps. Thus, the possibility of the formation of G4s *in vitro* on the fixed-sheared chromatin and their enrichment during G4-ChIP cannot be ruled out. These G4s formed on fixed-sheared chromatin would not represent the G4 landscape in the cells and yet explain the similarity we observed between the G4 DNA-IP^*nat*^ and G4-ChIP datasets. It thus becomes impossible to segregate chromatin-derived G4s from possible *in vitro*-derived G4s in the G4-ChIP data. These results also indicated other possibilities: if the single-stranded DNA could rapidly fold into G4s *in vitro* (G4 DNA-IP^*denat*^), could this also happen in the chromatin? Do such G4s form readily on the DNA in chromatin and are they physiologically relevant? These questions could be answered only if the G4s were captured solely from the chromatin without any possibility of contamination from G4s formed after fixation and shearing.

To clearly establish the G4 landscape under the constraints of the nuclear environment, it was important to first generate the quadruplex landscape in cells with a minimum *in vitro* artifacts. We worked on eliminating the possibility that some sequences, which do not exist as quadruplexes in live cells, adopt quadruplex structures during various stages of the G4-ChIP protocol post-fixation.

### A method for capture of G4s genome-wide without *in vitro* interference

**Figure 2:**
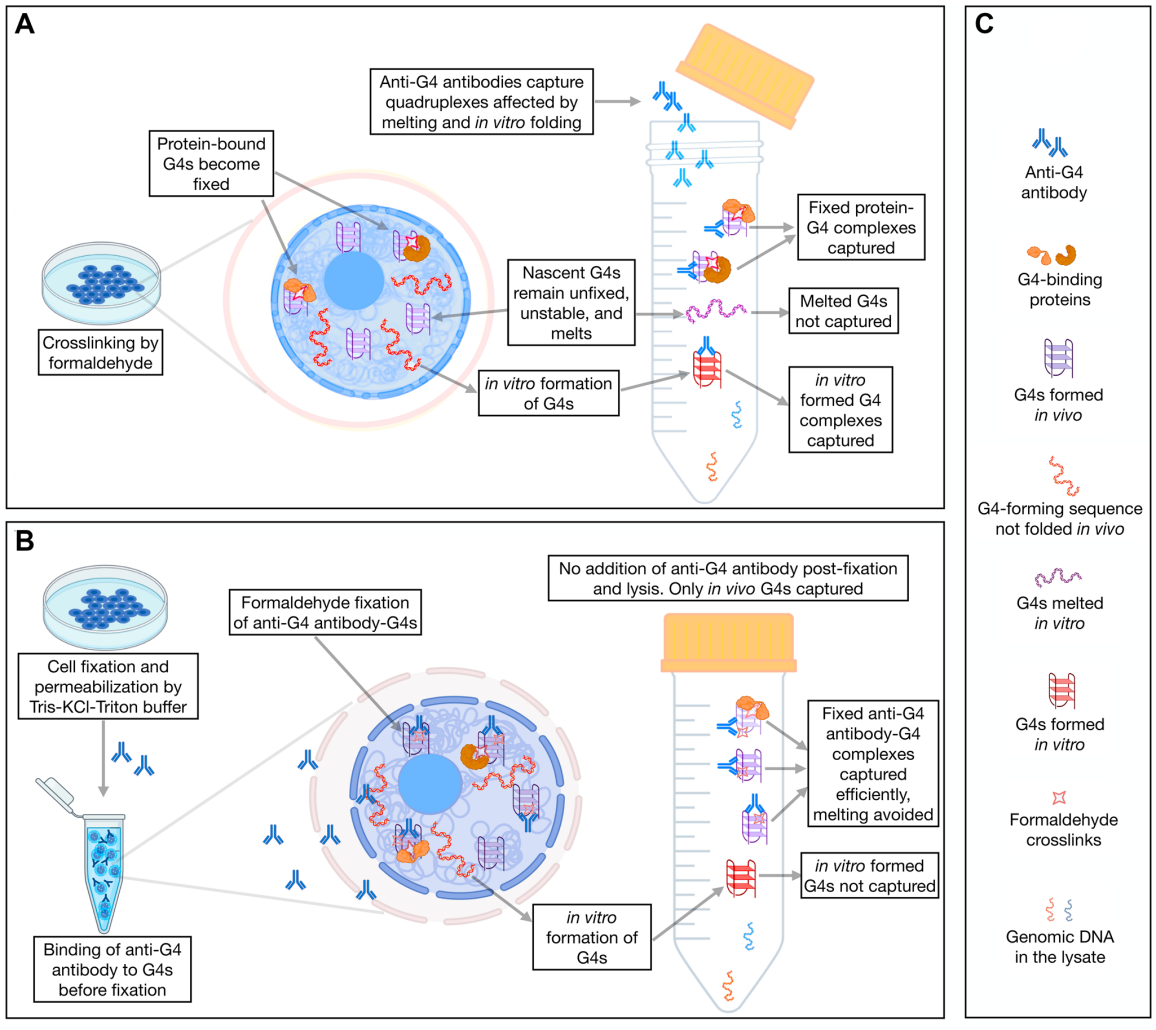
A comparative analysis of G4 landscape in cultured cells using *AbC* G4-ChIP. (**A**) A schematic of the conventional G4-ChIP highlighting the possibilities of false positives and negatives. (**B**) A schematic of the *AbC* G4-ChIP protocol highlighting the key differences from the conventional G4-ChIP and minimization of false positives and false negatives. (**C**) Character legend for A and B.

The G4-ChIP protocol relies on the fixation of G4-protein complexes by formaldehyde followed by lysis of the cellular nuclei, sonication and target precipitation using an antibody of interest. The capture of the G4s by this method depends on (i) an assumed interaction of the quadruplexes with proteins required for formaldehyde fixation and (ii) that the binding of the anti-G4 antibody is not sterically hindered by proteins covalently fixed onto the quadruplexes. Together these factors are expected to create false negatives in the G4 landscape identified by G4-ChIP (Figure 2A). Additionally, the melting effect of formaldehyde on nascent DNA segments [34, 35] of the sonicated chromatin creates single-stranded DNA capable of forming false positive G4s. Moreover, G4s seem to form rapidly and spontaneously without any need for DNA denaturation. These latter types of G4s can create false positives in the G4-ChIP data. To overcome these limitations, we developed a variation of the G4-ChIP to minimize these false positives and false negatives.

HEK293T cells were pretreated with formaldehyde briefly, followed by incubation with a buffer containing Triton-X 100 for permeabilization and KCl for maintaining/stabilizing the G4s in their native states (Figure 2B, 2C). These permeabilized cells were bathed with 1H6 antibody for two hours at room temperature. The antibody-bound G4s were fixed with formaldehyde ensuring that the quadruplexes were captured as antibody-G4 complexes, thereby minimizing false negatives. Also, no further addition of the antibody during the rest of the protocol ensured that any quadruplexes formed post-fixation were not captured. We call this new protocol *A*nti*b*ody *C*apture G4-ChIP (*AbC* G4-ChIP, detailed protocol is mentioned in methods). The DNA captured using *AbC* G4-ChIP was expected to enrich only the nuclear G4s. Due to the stabilizing effect of antibody-binding on G4s that would form transiently, the *AbC* G4-ChIP protocol allowed the capture of G4s as they formed in the nuclei, thereby generating a wider repertoire of G4s.

This *AbC* G4-ChIP sample (called CT^*nuc*^) was sequenced (Supplementary Table 6) and compared against the *in vitro* G4 DNA-IPs and G4-ChIP data (Supplementary Table 1). The G4-ChIP and G4 DNA-IP^*nat*^ co-clustered with each other, whereas the CT^*nuc*^ formed an independent cluster (Supplementary Figure 4A), suggesting that the *AbC* G4-ChIP returns a G4 landscape substantially different from either of the *in vitro* G4 DNA-IPs (heat-denatured or not) and the G4-ChIP (Supplementary Figure 4B). To further understand how CT^*nuc*^ was related to G4-ChIP and *in vitro* G4 DNA-IPs, we compared the signals of these datasets on their peaks reciprocally (Supplementary Table 2). The CT^*nuc*^ signals were distinctly observed at the G4 DNA-IP^*nat*^ and G4 DNA-IP^*denat*^ peaks (Supplementary Figure 5A, 5B, respectively). Overlapping subsets of CT^*nuc*^ peaks were rich in signals from G4 DNA-IP^*nat*^ as well as G4 DNA-IP^*denat*^ (Supplementary Figure 5C, 5D, respectively). While CT^*nuc*^ signals were abundant at G4-ChIP peaks, the G4-ChIP signals were poor at CT^*nuc*^ peaks (Supplementary Figure 6A, 6B, respectively). Very few CT^*nuc*^ peaks showed intersects with G4 DNA-IP^*nat*^ peaks (Supplementary Figure 7A), whereas nearly half of CT^*nuc*^ peaks showed overlaps with the G4 DNA-IP^*denat*^ peaks (Supplementary Figure 7B); overlaps between peaks of G4-ChIP and CT^*nuc*^ were also relatively weaker (Supplementary Figure 7C, 4B). The peaks were also compared by Jaccard indices (Supplementary Figure 8). These indices showed that the small number of G4 DNA-IP^*nat*^ peaks or the far numerous G4 DNA-IP^*denat*^ peaks had similar overlaps with the CT^*nuc*^ peaks (Supplementary Table 2). The G4-ChIP peaks, which overlapped strongly with *in vitro* G4 DNA-IP^*nat*^ peaks, showed a very weak intersection with CT^*nuc*^ peaks. A Venn diagram showing peak intersects for the four samples summarizes these properties of the peaks (Supplementary Figure 9A). Functional annotation of these peaks using *ChIPseeker* showed that the majority of G4s were expectedly captured from intergenic and intronic regions. Interestingly, only a small fraction of G4s were derived from immediate promoters (<1 kb proximal regions to transcription start sites) (Supplementary Figure 9B). We also analyzed the presence of known G4-forming sequence properties in these peaks. The (G_3_-N_(1-7)_)xn content was slightly different between CT^*nuc*^ and G4-ChIP (Supplementary Table 4). However, the GGG/CCC content between the G4-ChIP and CT^*nuc*^ peaks was strikingly different (*p*-values < 0.0001) (Supplementary Figure 3). Unlike G4-ChIP, the CT^*nuc*^ peaks were almost devoid of the low GGG/CCC content reads, a feature associated with the spontaneously forming G4s in the G4 DNA-IP^*nat*^. A *de novo* DNA sequence motif identification in the peaks from the four datasets also showed that *AbC* G4-ChIP pulls down G4s from regions with a highly significant presence of GGG-containing motifs (Supplementary Figure 10).

These properties of the CT^*nuc*^ sequence data showed that the *AbC* G4-ChIP protocol captures G4s of both kinds as represented by G4 DNA-IP^*nat*^ and *in vitro* G4 DNA-IP^*denat*^. In *AbC* G4-ChIP however, the spontaneously-forming G4s at GGG/CCC-poor regions account for a far lower fraction of the total G4s as compared to that in G4-ChIP. In contrast, G4-ChIP captures almost exclusively the G4s represented by G4 DNA-IP^*nat*^ and fails to capture the ones represented by G4 DNA-IP^*denat*^. Overall, the overwhelming similarities between G4-ChIP and *in vitro* G4 DNA-IP^*nat*^ show that G4-ChIP captures spontaneously forming G4s at low GGG/CCC sequences, including the G4s that form post-fixation. A comparison with CT^*nuc*^ showed that these low GGG/CCC quadruplexes are not available for antibody binding in intact cells. These are likely *in vitro* artifacts captured in G4-ChIP but eliminated in CT^*nuc*^.

A measure of this artifact was derived by analyzing G4 signals captured in these assays at randomly drawn genomic regions (details in the methods). Unlike the regions captured in these assays using the 1H6 antibody (where G4 signals were expected), these randomly selected regions were not expected to be rich in G4 signals. As expected, the G4 signals in these random genomic regions captured in CT^*nuc*^ were very low; across all the samples, about 78-83% of the regions contained just 0.001% of all the G4 signals captured (Supplementary Figure 11A). However, strikingly, the largest deviation from low or no signals in these random regions was observed in G4-ChIP and G4 DNA-IP^*nat*^ (Supplementary Figure 11A). When normalized for sequencing depth, the G4 signals in these randomly drawn regions were highest in G4 DNA-IP^*nat*^ followed by G4 DNA-IP^*denat*^, G4-ChIP and CT^*nuc*^ (Supplementary Figure 11B–11E, respectively) in decreasing order. In previously published G4-ChIP datasets also we observed a higher signal in randomly drawn regions than in CT^*nuc*^ (Supplementary Figure 12). The overall low levels of signals at randomly drawn regions showed that the 1H6 antibody does not capture DNA randomly, and the pull-down has specificity. The remarkable difference in the G4 signals between G4-ChIP and CT^*nuc*^ reinforced the fact that (i) G4s form spontaneously at many regions in the genome that can be drawn in any randomly chosen regions of the genome, (ii) the chromatin environment prevents the formation of these G4s, and (iii) between G4-ChIP and CT^*nuc*^, the latter is better at not capturing G4s formed at these randomly drawn regions thereby delivering a cleaner G4 landscape from the chromatin. Thus, the formation of G4s at random regions gives rise to artifacts similar to the G4 DNA-IP^*nat*^ and G4-ChIP. The formation of G4s at random regions in G4-ChIP likely happens during the antibody incubation step.

### An application of AbC G4-ChIP identifies CGGBP1 as an agent of constraints of the nuclear environment on G4 formation

**Figure 3:**
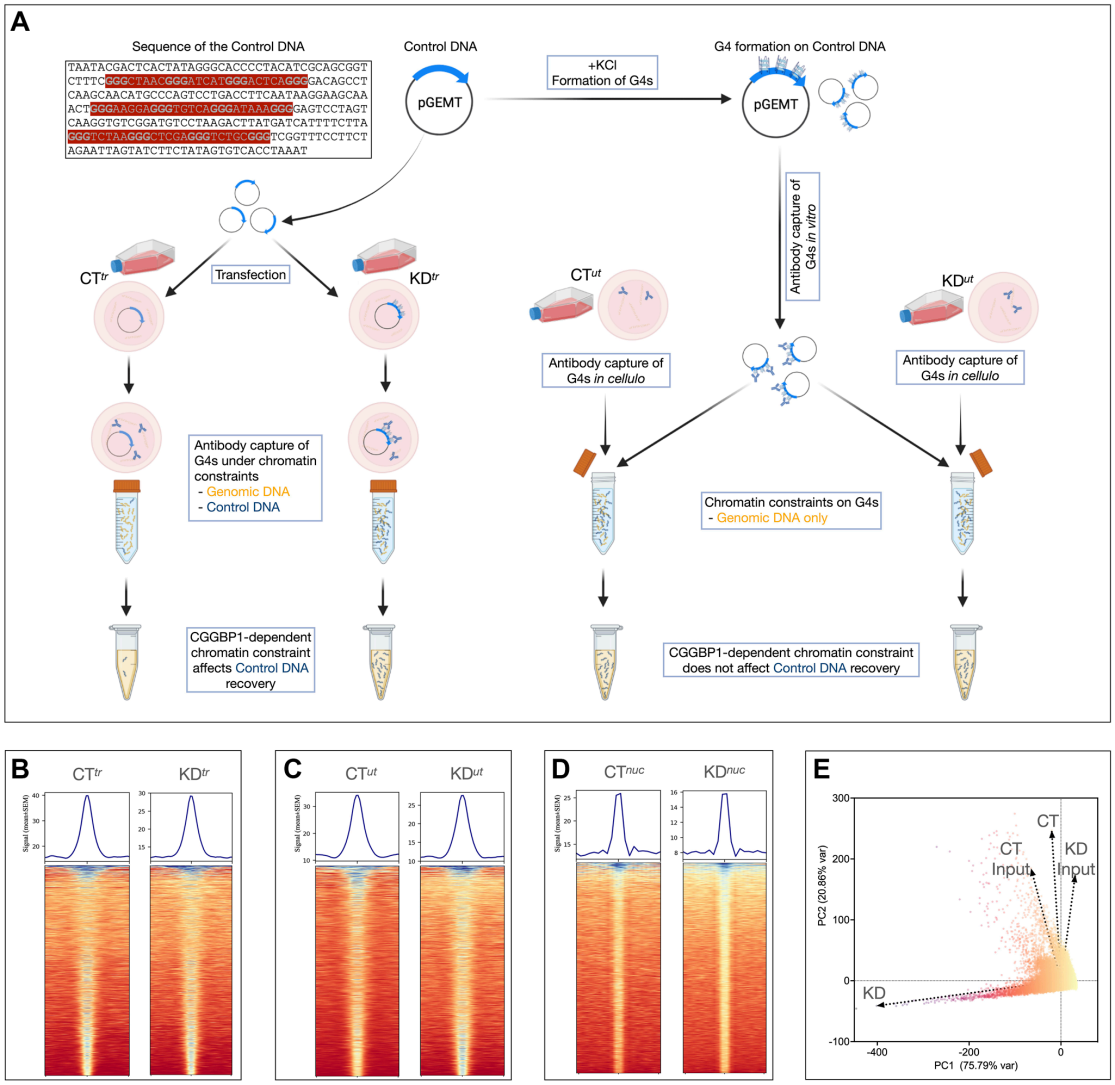
Validation of the *AbC* G4-ChIP protocol using a control DNA and its application to a CGGBP1 knockdown system. (**A**) A schematic of the control DNA (sequence with G-triplets highlighted in bold) cloned in pGEM-T Easy vector and its usage in ***AbC*** G4-ChIP. The left flank shows the protocol in which the control DNA (along with the carrier DNA) is transfected (samples CT^*tr*^ and KD^*tr*^). The right flank shows the samples CT^*ut*^ and KD^*ut*^ in which the preformed G4s on the control DNA are added to the lysates. As indicated, the effects of the intranuclear environments of live cells on G4s would be seen only in CT^*tr*^ and KD^*tr*^ but not in CT^*ut*^ and KD^*ut*^. (**B**–**D**) Heatmaps and signal plots showing G4 enrichment at peak regions as compared to the neighboring 5 kb flanks. The signals were consistently weaker at the peaks in KD samples as compared to the respective CT samples. (**E**) A PCA plot of the combined signals of the three CT and KD experiments shows that CGGBP1 depletion remarkably alters the G4 landscape. The values used for PCA loading were derived from the summation of signals in synchronous 0.2 kb genomic bins after the removal of bins with signals <3 and >100.

To test if *AbC* G4-ChIP actually eliminates false positive quadruplexes, we applied additional controls to differentiate the *in cellulo* G4s from the *in vitro* formed G4s confidently. We generated a Control DNA that contained three repeats of the unit canonical (*G*_3_ *-N*_5_ *-G*_3_ *-N*_5_ *-G*_3_ *-N*_5_ *-G*_3_) signature sequence (Figure 3A) and cloned it in the pGEM-T Easy vector. The quadruplex-forming property of a unit (*G*_3_ *-N*_5_ *-G*_3_ *-N*_5_ *-G*_3_ *-N*_5_ *-G*_3_) signature sequence (labeled DG in Supplementary Figure 13) was established using CD spectroscopy under different concentrations of KCl. DG showed a positive peak around 210 nm in the CD spectra (Supplementary Figure 13A). The negative peak around 240 nm and the positive peaks at 260 nm and 290 nm further confirmed the formation of hybrid G4s [53, 54]. This G4-forming property of DG was lost when it was paired with its reverse complementary oligonucleotide in a Watson-Crick pairing (labeled WC in Supplementary Figure 13). The hypochromic thermal melting curve (at 260 nm) observed for DG was independent of the KCl concentration, indicating the formation of G4s [55, 56] (Supplementary Figure 13B). Electrophoretic mobility shift assays further indicated that the hybrid G4 conformations preferred by DG were intramolecular (Lanes 2–6 in Supplementary Figure 13C) as it moved faster than the d(T)_27_ oligonucleotide (Lane 1 in Supplementary Figure 13C). We could conclude from these CD and EMSA experiments that the Control DNA could form an intramolecular hybrid G4 which can have any of the quadruplex folds depicted in Supplementary Figure 13D. Subsequently, this Control DNA was used in the *AbC* G4-ChIP protocol as a spike-in to distinguish G4s formed only under constraints of the nuclear environment from those formed only *in vitro*.

This Control DNA could be used in two different ways: (i) added to the sonicated lysate to represent the *in vitro* false-positive quadruplexes formed without any constraints of the nuclear environment, or (ii) transfected into the cells so that the quadruplexes formation on the same sequence would be subjected to constraints of the nuclear environment (Figure 3A). The differential capture of the Control DNA in the two samples would reveal the extent of constraints of the nuclear environment on G4s formation on sequences with canonical G4s forming signatures. We applied the *AbC* G4-ChIP with the Control DNA in these two different ways for functional studies on a candidate G4-regulatory protein CGGBP1.

CGGBP1 is a GC-rich DNA triplet repeat-binding protein. It exhibits a heightened occupancy at putatively G4-forming GGG triplets in growth-stimulated cells (as described elsewhere [48] and Supplementary Figure 14). We hypothesized that if the possibility of constraints of the nuclear environment by CGGBP1 is true, then the depletion of CGGBP1 would affect the G4 landscape. *AbC* G4-ChIP with the Control DNA was employed to determine the G4 landscape in HEK293T cells with or without CGGBP1.

CGGBP1 was knocked down in HEK293T cells (Supplementary Figure 15) as described elsewhere [5]. The non-targeting shRNA sample was called CT, and the CGGBP1-depleted sample was called KD. The CT and KD samples were transfected with the Control DNA and subjected to *AbC* G4-ChIP (Figure 3A). To rule out any transfection efficiency bias between CT and KD samples due to secondary structure formation in the Control DNA, we co-transfected an equimolar amount of a carrier DNA, with no detectable G4-forming sequence (all tetrad-forming GGG sequences replaced with ATG; *allquads* output, not shown), also cloned in pGEM-T Easy vector. The recovery of the Control and the carrier DNA from the inputs were verified by quantitative PCR (Supplementary Figure 16). There was no significant difference in the transfection efficiency between the CT and KD. These samples were called CT^*tr*^ and KD^*tr*^. In these samples, the Control DNA was expected to be captured with 1H6 antibody only if it formed quadruplexes in the nuclei, presumably under constraints of the nuclear environment. In parallel, CT and KD were also processed for *AbC* G4-ChIP without any transfections of either Control or carrier DNA (CT^*ut*^ and KD^*ut*^, respectively). These samples were processed till nuclear lysis without any transfection. Then, an equimolar mix of Control and carrier DNA was allowed to fold *in vitro* in the presence of KCl, incubated with 1H6 antibody, formaldehyde-fixed and added to the lysates of CT^*ut*^ and KD^*ut*^ (Figure 3A). The CT^*tr*^, KD^*tr*^, CT^*ut*^ and KD^*ut*^ samples were processed identically for the rest of the *AbC* G4-ChIP protocol and sequenced. In addition, we also sequenced the *AbC* G4-ChIP pull-down from a KD sample without any transfection or *in vitro* addition of Control or carrier DNA to capture only the nuclear G4s. This sample (called KD^*nuc*^) was compared against the CT^*nuc*^ sample described above (Figure 2 and Supplementary Table 7). Thus, *AbC* G4-ChIP was performed on three non-identical replicates of CT (CT^*nuc*^, CT^*tr*^ and CT^*ut*^) and KD (KD^*nuc*^, KD^*tr*^ and KD^*ut*^).

First, we analyzed the Control DNA pull-down in CT^*tr*^, KD^*tr*^, CT^*ut*^ and KD^*ut*^ datasets. The coverage of sequencing reads on the Control DNA in the input samples were low, with an enrichment in the *AbC* G4-ChIP samples. The number of reads mapping to the Control DNA increased upon CGGBP1 knockdown in KD^*tr*^ compared to CT^*tr*^ (Supplementary Table 8). The read counts mapping to the Control DNA in CT^*ut*^ and KD^*ut*^ remained unaffected. In CT^*tr*^, the Control DNA was captured as only six reads, which increased to 390 reads in KD^*tr*^. Linked to the Control DNA sequences, the vector backbone also showed similar variations (not shown). It could be concluded from these results that upon transfection, quadruplex formation on the Control DNA was under an inhibitory influence of CGGBP1. Depletion of CGGBP1 relieved this inhibition on the Control DNA, including the linked vector backbone. The capture of G4s similarly between CT^*ut*^ and KD^*ut*^ samples and very strongly only in KD^*tr*^ also established that the *AbC* G4-ChIP captures the G4s efficiently and specifically in a quantitatively verifiable manner. Thus, the differences in G4s prevalence on the Control DNA between CT^*tr*^ and KD^*tr*^ could represent the influence of CGGBP1 on G4 formation throughout the genome in the constraints of the nuclear environment. We analyzed the genome-wide G4 landscape captured in the three CT and KD samples and asked if, just like on the Control DNA, the constraints of the nuclear environment exerted by CGGBP1 are also observed on the genomic sequences.

G4 enrichment in CT (CT^*nuc*^, CT^*tr*^, CT^*ut*^) and KD (KD^*nuc*^, KD^*tr*^, KD^*ut*^) was confirmed using peak calling and validated by calculating the read density within the peaks compared to the flanking regions (Figure 3B–3D). The three samples also correlated well, such that centrally enriched G4 signals were observed for each dataset pair (Supplementary Figure 17). We observed a generally slightly weaker enrichment at the peak centers in KD samples as compared to the respective CT samples (Figure 3B–3D). This lower concentration of G4s at the peak regions suggested that interspersed *de novo* quadruplexes were formed and captured at non-peak regions or more peaks upon CGGBP1 depletion (number of peaks indicated in Figure 3B–3D). A principal component analysis of the pooled CT and KD datasets and the respective inputs showed that CGGBP1 depletion affected the G4 landscape drastically and accounted for the largest variance (Figure 3E).

**Figure 4:**
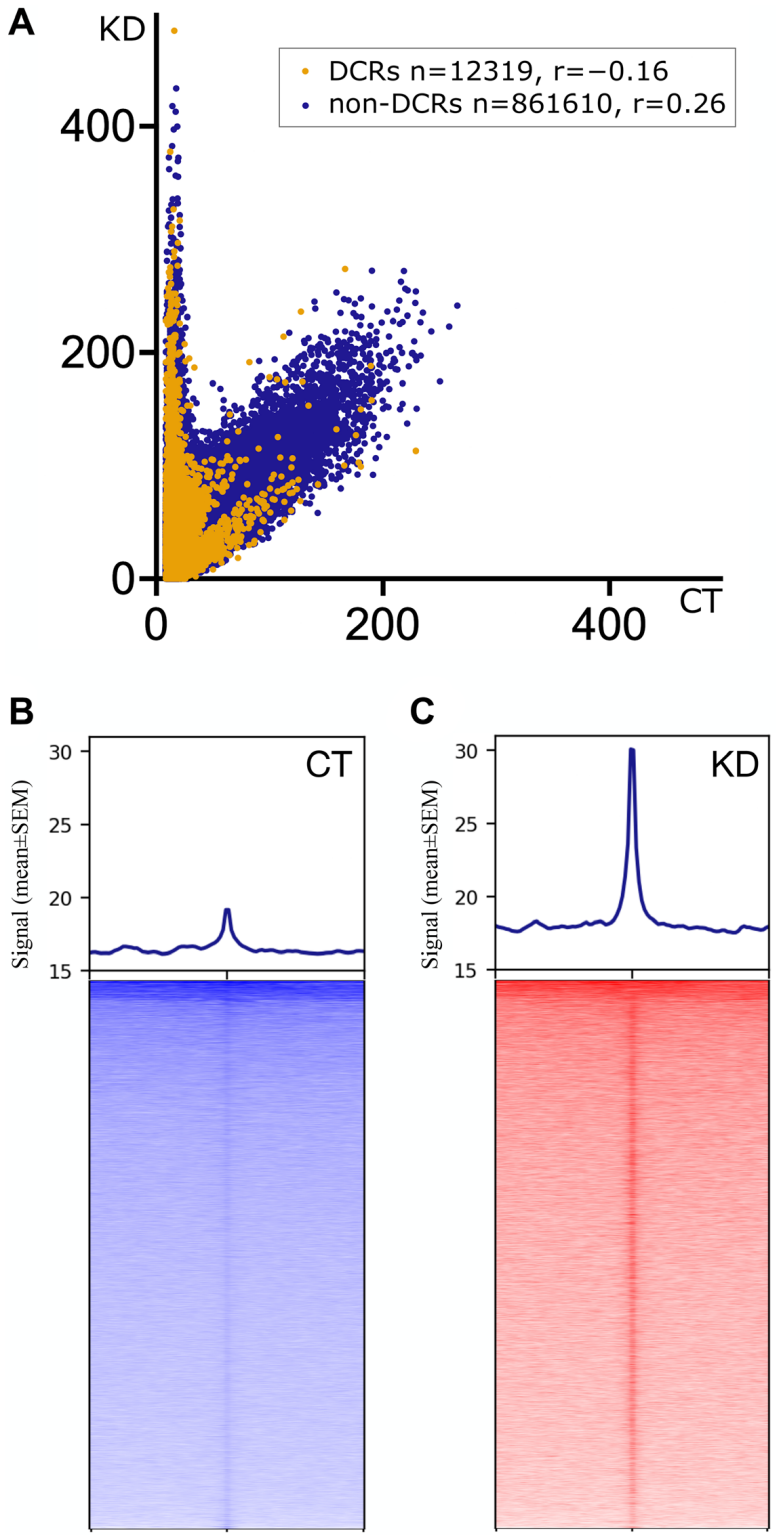
Distribution of G4 signals as captured in pooled CT (CT^*tr*^, CT^*ut*^ and CT^*nuc*^) and KD (KD^*tr*^, KD^*ut*^ and KD^*nuc*^) samples. (**A**) The correlation shows the abundance of G4 signals in the 0.2 kb bins genome-wide (*n* = 873929). Most 0.2 kb bins show no quantitative difference in the signal (r = 0.26), as represented by the cluster of non-DCRs (dark blue dots). However, a small fraction of the 0.2 kb genomic bins (~0.01%) show a stronger presence of G4 signals in KD than in CT with a negative correlation (r = −0.16) represented by the DCRs (orange dots). The Y-axis represents the input-normalized signal for KD, and the X-axis represents the input-normalized signal for CT per 0.2 kb bins. The DCRs and non-DCRs are negatively correlated. The correlation values for DCRs (yellow dots) and non-DCRs (blue dots) are mentioned in the inset. (**B**, **C**) The pooled G4 signal in CT and KD show a qualitative difference on the 10 kb flanks from the center of the DCRs. In KD, the center of the DCRs carries higher enrichment of the signal than in CT. In general, the neighboring regions of the DCRs show weaker G4 signals in CT compared to KD. The heatmaps were generated using *deepTools plotHeatmap* with the adjusted Y-axis values for the signal to plot the profile (mean ± SEM). The DCRs in the heatmaps were sorted in the decreasing order of the signal from the top to the bottom of the plot.

To compare the G4 profiles of CT and KD, we first calculated signals in 0.2 kb bins genome-wide, selected bins with ≥3 (removal of no signal biases) and ≤100 reads (removal of PCR artifacts) in all the samples separately. These G4 signals were then corrected for regional representational biases through a normalization based on signals from the same regions in the respective inputs. Paired *t*-tests were performed independently for each pair of 873929 genomic bins from CT and KD (*n* = 3 pairs; CT^*nuc*^–KD^*nuc*^, CT^*tr*^–KD^*tr*^ and CT^*ut*^–KD^*ut*^). We discovered 12319 differentially captured regions (DCRs) with a significant difference in G4s between CT and KD samples (*p*-value < 0.01, Q:1%: two-stage linear step-up procedure of Benjamini, Krieger and Yekutieli; pairwise *p*- and *q*-values for each bin in Supplementary Table 9). A correlation analysis between pooled G4 signals in CT and KD (Figure 4A) showed two major categories of 0.2 kb bins; most bins showed no difference between CT and KD (non-DCRs), and the DCRs with a conspicuous increase in the signal in KD over CT (Figure 4A and Supplementary Figure 18). Signals from the three pairs of CT and KD in the flanks of these DCRs showed that they were rich in G4s with a net increase in G4 signal upon CGGBP1 depletion (Figure 4B, 4C). These were apparently the regions where CGGBP1 exerted constraints of the nuclear environment on G4 formation, and the depletion of CGGBP1 led to a net increase in G4 signals. Conspicuously, there were a much lower number of regions with a decrease in G4 formation in KD as compared to CT (Figure 4A and Supplementary Figure 18). We further analyzed the properties of these differentially captured regions (DCRs) where G4-formation is regulated by CGGBP1.

To understand the functional relevance of the DCRs, the proximity of the DCRs with a panel of functional genomic regions was calculated using (i) UCSC annotations for genes and CpG islands, (ii) FANTOM annotations for enhancers, and (iii) ChIP-seq data for markers of replication origins in human cells. There were no obvious spatial associations of the DCRs with any of these functionally annotated landmarks (Supplementary Figure 19). However, we could establish that in previously reported publicly available G4-ChIP datasets, there is a weak but specific enrichment of G4 signals at DCRs (Supplementary Figure 20), suggesting that the DCRs are recognized by 1H6 as well as BG4 antibodies. Candidate DCRs (Supplementary Figure 21) could be detected by PCR in G4-ChIP as well as *AbC* G4-ChIP (not shown). We argued that if the DCRs discovered by the application of the *AbC* G4-ChIP were indeed biologically relevant, they would exhibit features predictable by the known functions of CGGBP1. We analyzed the properties of these regions with reference to the features of DNA sequences associated with regulation by CGGBP1: interspersed repeats, GC-richness, G/C-skew and CTCF-binding sites [48, 50, 57, 58].

As the net capture of G4s at DCRs was increased due to CGGBP1 knockdown, it was a strong possibility that CGGBP1 occupancy on the DNA mitigates G4 formation. To test this possibility further, we employed a polymerase stop assay as described elsewhere [59] using the Control DNA and recombinant or immunoprecipitated CGGBP1 (detailed protocol in methods). The Control DNA formed stable G4s in the presence of KCl compared to LiCl (Supplementary Figure 22A). Pre-incubation of the Control DNA with recombinant CGGBP1 (rCGGBP1) caused a stronger polymerase stop in the presence of LiCl than KCl, resulting in poorer amplification in the presence of LiCl (Supplementary Figure 22B, 22C). Since G4-formation is relatively incompatible with LiCl, it could be concluded that the polymerase stop observed in the presence of LiCl was caused by rCGGBP1 occupying the Control DNA template in the absence of G4s. Such a polymerase-stopping effect by rCCGBP1 was not observed in the presence of KCl. We could recapitulate these findings by using CGGBP1 purified from HEK293T cells as well. Immunoprecipitated and freshly eluted CGGBP1 (Supplementary Figure 22D) under low salt conditions was used in the polymerase stop assays. A non-specific isotype immunoprecipitate was used as a negative control. Similar to the effect of rCGGBP1, the immunoprecipitated CGGBP1 also exerted polymerase stop in conditions where G4s are not favored (Supplementary Figure 22E). We could establish that even in the presence of KCl, where G4 formation on Control DNA is strongly favored, a pre-incubation with immunoprecipitated CGGBP1 reduced the amount of G4s that could be pulled down using the 1H6 antibody *in vitro* (Supplementary Figure 22E). Based on these results, we concluded that the presence of CGGBP1 hinders G4 formation through a mechanism that involves the interaction of CGGBP1 with unfolded G4-forming DNA. Such a mechanism could underlie the net increase in G4s upon CGGBP1 depletion observed at the DCRs.

**Figure 5:**
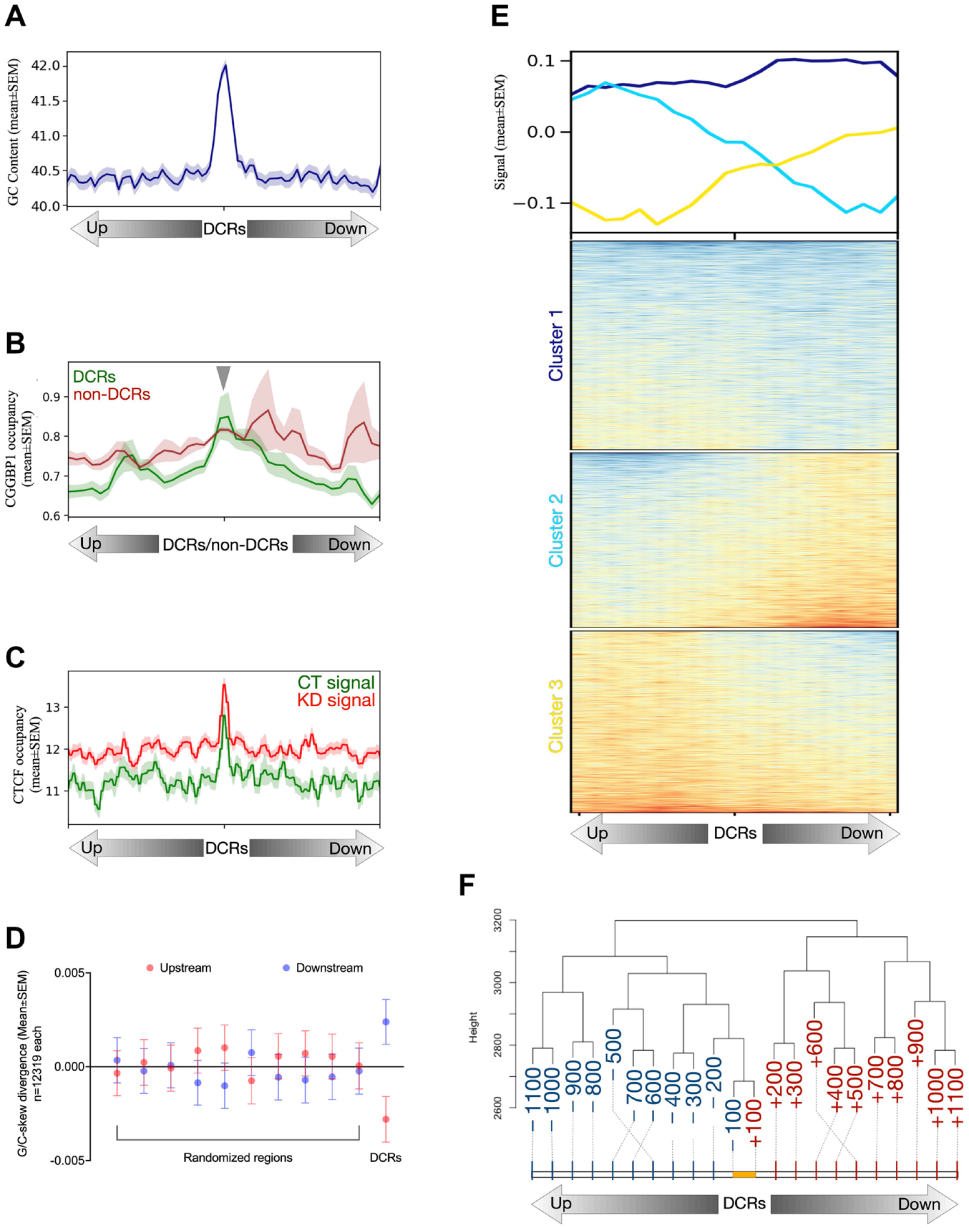
Features of the DCRs. (**A**) The DCRs show that these CGGBP1-regulated genomic regions have stronger GC content than their immediate flanks of 2 kb. The GC content signal is plotted as mean ± SEM, and the Y-axis represents the signal values. (**B**) The signal from genome-wide occupancy of CGGBP1 under growth stimulation (GSE53571) has been plotted on the 1 kb flanks from the center of DCRs and non-DCRs. The DCRs (green) show enrichment of CGGBP1 occupancy in their centers as compared to the non-DCRs (dark red) with a background level of the signal devoid of any specific enrichment. The lines were drawn as mean ± SEM, and the Y-axis shows the signal values. (**C**) The non-canonical CGGBP1-dependent CTCF binding sites show that upon knockdown of CGGBP1, the occupancy of CTCF on DCRs has heightened occupancy (dark red) than seen at normal levels of endogenous CGGBP1 (green). The signal is plotted in 10 kb flanks from the center of the DCRs with the lines representing the mean ± SEM of the signal and the Y-axis representing the signal values. (**D**) The DCRs show a quantitative stronger G/C-skew as measured in their 1 kb flanks compared to the same number of randomly expected genomic regions sampled 10 times. The randomly expected genomic regions show a similar difference in G/C skew on their 1 kb flanks as compared to the DCRs. (**E**) The heatmap shows a qualitative and asymmetric distribution of G/C-skew on the 1 kb flanks of the DCRs. The DCRs are clustered to show stronger changes in G/C-skew in the flanks (cluster 2 and cluster 3) as well as minimum to no change in the G/C-skew (cluster 1). The lines represent the mean signal of G/C-skew calculated for the DCRs. (**F**) The G/C-skew has been binned into 0.1 kb bins in 1 kb flanks of DCRs and forms distinct clusters. The upstream and downstream bins form separate clusters wherein the respective bins cluster differently with the proximal bins. The yellow line represents the length of DCRs and the numbers represent the distance (0.1 kb) from the center of the DCRs either upstream (blue) or downstream (red). The Y-axis shows an arbitrary distance from the 0.1 kb bins. The dashed lines indicate the end of the 0.1 kb bins. The 0.1 kb bins are not drawn to scale. The cluster has been generated in R using the hierarchical method “manhattan”.

We hypothesized that the CGGBP1-binding sequences would be overrepresented in the DCRs compared to the non-DCRs and tested this using the CGGBP1 ChIP-seq data published elsewhere [48]. Short interspersed nuclear elements (SINEs), of which Alu elements are the most prominent binding sites for CGGBP1, were indeed observed at a lower level at DCRs than that expected from the genomic average or the non-DCRs (Supplementary Table 10). Such a difference was not observed for LINE elements, another major interspersed repeat regulated by CGGBP1 but not its major binding site. The DCRs where G4 formation was mitigated by CGGBP1 were expectedly rich in GC content (Figure 5A) as Alu elements are concentrated in GC-rich segments of the genome. Since the binding of CGGBP1 was expected to correlate oppositely with G4 formation, we expectedly observed a very weak CGGBP1 occupancy at any population of G4s, including DCRs as well as non-DCRs (Figure 5B). At the DCRs, CGGBP1 occupancy showed a weak but central enrichment, suggesting that these regions are in flux between ‘CGGBP1-bound G4-free’ or ‘CGGBP1-unbound G4-formed’ states. These results were striking as the CGGBP1 occupancy data were derived from a different cell line and suggest that the formation of G4s is a widespread property of DNA even under chromatin constraints, and CGGBP1 is similarly deployed in various cell types to mitigate G4 formation.

G/C-skew is a property of DNA regions regulated by CGGBP1 [50]. This includes non-canonical CTCF-binding sites described in HEK293T cells at which CTCF occupancy depends on CGGBP1 [5]. The DCRs were also rich in CTCF-binding sites (Figure 5C). However, the CTCF-binding was observed at comparable levels in non-DCRs as well (not shown). It seems that the CTCF occupancy coincides with G4 prevalence and the regulation of CTCF occupancy or G4 formation by CGGBP1 may be acting through independent mechanisms. A G/C-skew calculation showed that the DCRs, including 1 kb flanking regions, had an unexpectedly high G/C-skew (Figure 5D). Interestingly, the G/C-skew was asymmetrically distributed across the DCRs (Figure 5E). The upstream and downstream 0.1 kb regions spanning in the 1 kb flanks were segregated into separate clusters (Figure 5F) due to unexpectedly high transitions of G/C-skew at DCRs.

Collectively, our findings showed that using *AbC* G4-ChIP, we could reliably capture and identify G4s genome-wide from the cells. *AbC* G4-ChIP minimizes the capture of false positive G4s (the population most common between G4-ChIP and G4 DNA-IP^*nat*^). It also captures a previously unreported set of G4s (the population most common between G4 DNA-IP^*denat*^) and thus eliminates false negatives. We could apply this method to HEK293T cells with and without the endogenous levels of CGGBP1 (CT and KD, respectively) and discover regions where mitigation of G4 formation by CGGBP1 coincides with unexpected G/C-skew and CGGBP1-regulated CTCF occupancy.

## DISCUSSION

G4s are some of the most well-studied DNA secondary structures. Decades of *in vitro* studies have defined the chemical environment (presence of KCl) in which the quadruplexes can form on some canonical sequences. Very important and lasting conclusions about the nature of bonding leading to the formation of G-tetrads and the resultant formation of quadruplexes have been generated using *in vitro* assays. A combination of G-triplets separated by spacers has long been considered model sequences for G4 formation. Many computational tools rely on such sequences to report potential quadruplex formation. However, two major advances have challenged the restriction of quadruplexes to such canonical sequences. First, the antibodies such as 1H6 detect G4s formed by a variety of T and G combinations not limited to the model sequences. Second, characterization of G4s genome-wide using the anti-G4 antibody BG4 does not show any restriction of quadruplexes to the canonical sequences. In fact, these studies underscore the complexity of DNA secondary structure landscape in cellular nuclei.

Simplistically, it is expected that the G4-ChIP sequencing datasets are likely to have signature sequences at peak centers, using which a G4 consensus sequence can be derived. However, such a signature sequence for G4s in G4-ChIP datasets has remained elusive. This is reaffirmed by the findings reported elsewhere [33] as well as by running the G4-ChIP sequencing data through programmes such as *allquads*. The description of G4 landscapes from chromatin available in the literature so far suffers from a lack of calibration of the G4 potential of the genomic DNA. After all, G4 formation is a property of the DNA not necessarily contingent upon any chromatin component other than the DNA itself. Calibration of the G4 potential of the genomic DNA would give a reference frame of the possible G4s in the genome against which the chromatin-derived G4 landscape could be compared. In the absence of such a calibration of the G4 potential of the genome, it becomes difficult to gauge the completeness and genuineness of the chromatin-derived G4 landscape. This is especially important because unlike the ChIP-based characterization of DNA-protein interactions, the G4 landscape characterization from cells is not based on a presumed DNA-protein interaction and yet employs the same biochemical principles in the G4-ChIP protocol. The G4s are expected to be captured in G4-ChIP if they survive the primary treatments of the chromatin with formaldehyde, detergents and sonication (proneness to false negatives) or form *de novo* (proneness to false positives) during these steps. A comparison against the total G4 potential of the human genomic DNA allows a more objective evaluation of these two types of errors in G4 landscapes captured from the chromatin.

The two *in vitro* DNA-IPs described in this study provide a G4 calibration potential for human genomic DNA. Although we performed these assays on DNA purified from HEK293T cells, the G4 potential of human DNA shown by these assays is expected to be near-universal for any human DNA except for any effects of DNA chemical modifications, such as cytosine methylation, on G4 formation. Interestingly, we found two different types of G4s: one which was formed by spontaneously overcoming the double strandedness at 4°C and another one which was formed readily when the strand separation is facilitated, as we achieved through heat denaturation. Expectedly, the second type of facilitated G4s was widespread, G/C-rich and consisted of the spontaneously forming G4s as a subset, whereas the spontaneously forming G4s were limited and relatively G/C-poor. By comparing against this superset of possible G4s that can form in the human genomic DNA, the following possibilities can be tested for any chromatin-derived genome-wide G4 landscape: What fraction of the possible G4s form *in vivo* on the chromatin? What fraction of the chromatin-derived G4s are spontaneous or facilitated types of G4s? Is G4 formation in the chromatin facilitated such that the G4s form in the chromatin beyond the *in vitro* determined G4 potential of the DNA, or is there a mitigation of G4 formation in the chromatin as proposed elsewhere [52]? In order to answer these questions precisely, we must be able to compare an absolutely *in vitro* generated G4 potential of genomic DNA against an absolutely chromatin-derived G4 landscape with no possibility of any false positives or false negatives. Our description of the *AbC* G4-ChIP alongside the *in vitro*-derived G4 potential provides a powerful dataset to understand the scope and nature of G4 formation in chromatin that is largely free of false positive and false negative G4s. We observe that the spontaneously forming G/C-poor G4s are prevalent in G4-ChIP but nearly absent from *AbC* G4-ChIP, showing a reduction in the false positive G4 capture. This reduction of spontaneously forming G4s in *AbC* G4-ChIP is due to the elimination of any post-fixation and sonication incubation with the antibodies. Also, the limited but larger presence of G/C-rich facilitated G4s in the *AbC* G4-ChIP as compared to that in G4-ChIP shows the elimination of false negative G4s. This larger scale of G4 capture in *AbC* G4-ChIP is due to the usage of antibodies for the stabilization of G4s formed in the chromatin. In the G4-ChIP protocol, such G4s would either escape crosslinking if not bound by proteins or escape capture if protein-bound due to steric hindrances to G4-antibody binding.

The disconnect between our knowledge from *in vitro* studies on G4s and the complex G4 landscapes discovered in cells also leads to questions central to G4 biology: Are there sequence properties other than (G_3_-N_(1-7)_)xn that form the quadruplexes as well? Are there different mechanisms to regulate G4 formation at different types of sequences? Are there protein regulators of G4 formation? What are these proteins? Do they work in a sequence-dependent manner? Some of these questions can be answered by analyzing G4 landscapes and their sequence properties with or without perturbation of putative G4 regulatory genes. Despite our knowledge of multiple G4-binding proteins, such targeted studies to dissect subtypes of quadruplex-forming sequences based on their dependence on putative regulator genes have not been reported. The innate nature of cross-strand annealing combined with repetitive sequences in the human genome makes the DNA prone to randomly adopting multiple conformations. This problem is further exacerbated by the sheer size of the human genome, the restricted nuclear volume and the need to reset the genomic DNA arrangement in the nuclei through replication and transcription [60, 61]. Obviously, this challenge could not be addressed just by the regulators of higher-order chromatin conformation with their sequence-specific DNA bindings. In agreement with this, the highly conserved histone proteins, related protein machinery dedicated to packaging DNA into chromatin and single-strand DNA-binding proteins exhibit little DNA sequence preferences [20, 62]. These mechanisms disallow the existence of nascent DNA, which would be prone to adopting multiple conformations as allowed biochemically and thermodynamically in the nuclear environment. Collectively, these mechanisms constitute constraints of the nuclear environment on the property of genomic DNA to adopt secondary structures.

Our findings using the *AbC* G4-ChIP and Control DNA show that G4 formation *in vitro* is different from the G4 landscape in the cellular nuclei. While the *in vitro* G4 formation is unconstrained, the nuclear environment presents conditions which mitigate G4 formation. The *AbC* G4-ChIP captures the cellular G4 landscape with a discernible reduction in *in vitro* artifacts as compared to the conventional G4-ChIP protocol.

The application of the *AbC* G4-ChIP protocol to a CGGBP1 knockdown system also allows us to query the quadruplex formation and changes therein show overlaps with the sequences previously reported to be regulated by CGGBP1. This validatory detection of differential G4 formation at sequences with properties previously reported for regulation by CGGBP1 shows that the *AbC* G4-ChIP protocol is reliable and reports the quadruplexes such that the data are interpretable with reference to previously reported independent experiments. The *AbC* G4-ChIP avoids the capture of the G4s formed rapidly *in vitro* during the incubation steps, such as the incubation with the antibody. The regulation of G4 by any gene can be best studied only if there is a broad capture of G4s with no *in vitro* background. Functional studies aiming to find G4-regulatory genes, such an *in vitro* background as returned by G4-ChIP would confound conclusions as it is independent of any biological features of the samples and depends solely on DNA strandedness and sequence properties. The *AbC* G4-ChIP protocol offers a precise and identification of G4s from the nuclei with a greater diversity of G4s allowing its usage in G4 regulatory functional studies more reliably. We also discover a generic and widespread sequence property of interstrand G/C-skew that makes the DNA prone to G4 formation. With all the advantages *AbC* G4-ChIP offers over the conventional G4-ChIP, it is a protocol that takes longer to perform, the initial steps of permeabilization and antibody-capture are very sensitive and require nearly 5–10 times more antibody. For subsequent sequencing of the purified *AbC* G4-ChIP DNA, it is likely that pooling of samples from multiple runs will be needed.

Our analysis also reveals that the *allquads* G4 signature is strongly associated with *in vitro* formation of these structures. The G4 formation in the cellular nuclei is relatively less dependent on the (G_3_-N_(1-7)_)xn signature. We did not find any sequence property of G4s enriched in the chromatin-derived G4s using *AbC* G4-ChIP as compared to the *in vitro*-derived G4s. These findings suggest that G4 formation is a biochemical feature of the DNA with no evolutionary selection for stabilization of long-term facilitation of G4. This conclusion is supported by the fact that there are no known G4-binding domains or proteins which would bind to and stabilize G4s. The complex chromatin organization of nuclear DNA would benefit by preventing G4 formation and resorting to resolving them by recruiting helicases only when the prevention of G4 formation fails. It follows that protein binding to G4-forming sequences and capture of G4s at the same regions are hence mutually exclusive, a possibility supported by our observations of poor CGGBP1 occupancy at G4s.

We observe that the constraints of the nuclear environment discretize as well as restrict G4 formation. This is reflected in the number of peaks, which is larger in CT^*nuc*^ than in G4 DNA-IP^*nat*^ but less than in G4 DNA-IP^*denat*^. In the absence of any heat denaturation, the G4 DNA-IP^*nat*^ retains its stable long-term G4 landscape with no significant *de novo* formation of G4s at random regions during the antibody incubation. Most importantly, the G4s captured in G4 DNA-IP^*denat*^ are a range of G4s that are formed upon a range of heat energy input to melt the DNA strands. Apparently, the regions with the highest energy thresholds form the most stable G4s and get identified as peaks. The possibility of overcoming such extreme energetic requirements is an impossibility in the nuclear environment. This suggests that the energetic constraints enforced by double stranded nature of the DNA and its sequence are a biochemical impediment to G4 formation in the nuclear environment. The inverse correlation between G4 DNA-IP^*denat*^ peaks and G4-ChIP or *AbC* G4-ChIP peaks demonstrates this. However, beyond the peaks, the G4 DNA-IP^*denat*^ data contains G4 enrichment which is present in *AbC* G4-ChIP peaks more than in G4-ChIP peaks. The nature of the constraints of the nuclear environment on G4 formation is expected to be widespread, given the hindrances to DNA processes imposed by the random formation of secondary structures. Clearly, there is a larger repertoire of helicases that resolve these secondary structures, including the G4s, than the possibility of some proteins with dedicated quadruplex stabilizing functions. The growing number of G4-interacting proteins does not indicate any such quadruplex stabilizing function, although a paucity of targeted studies tests this possibility.

Our study reports such a targeted test of function for CGGBP1 on G4 formation. CGGBP1 is not a G4-binding protein. Similarly, it is not established for the many G4-binding proteins if their binding is to the actual quadruplex structure or to the immediate vicinity of the structure. This argument is supported by the absence of any quadruplex-binding domain common to these proteins [24]. An additional set of proteins important in this context are those which can bind to the DNA sequences capable of forming G4s but not to the quadruplex structures per se. The known DNA-binding properties of CGGBP1 indicate that it is capable of binding to G/C-rich sequences with G-repeats [24, 48, 57, 58]. These sequences are strong candidates for G4 formation. The binding to quadruplex-forming sequences but not the quadruplexes per se is logically a feature of proteins, which through binding to the DNA, would preclude quadruplex formation. These proteins, such as CGGBP1, would be able to exert constraints of the nuclear environment on quadruplex formation. Our results show that CGGBP1 binds to sequences capable of G4 formation and exerts constraints of the nuclear environment on quadruplex formation. The weak CGGBP1 occupancy at sequences enriched as G4s reaffirms the notion that CGGBP1 binding could be an impediment to G4 formation. The DCRs, with their preference for CTCF occupancy, underscore the potential functions of G4-forming regions as chromatin boundary elements. The preponderance of G4s in the heterochromatin [63] and mitigation of G4 formation at CTCF-binding sites suggest that heterochromatinization, G4 formation and regulation by CGGBP1 are interlinked phenomena. Interestingly, our results highlight a property of quadruplex-forming DNA sequences, which is much less appreciated in G4 biology; G/C-skew. The *AbC* G4-ChIP presents a powerful technique to decipher the cellular G4 landscape and its regulation and it has the potential to be adapted for discovering any DNA secondary structures genome-wide against which reliable antibodies are available.

## METHODS

### Synthesis and cloning of the control and the carrier DNA

We have designed and procured a set of oligonucleotides (Supplementary Table 11) to generate the Control and the carrier DNA by PCR amplification. The synthesized fragments were gel-purified and cloned into the pGEM-T Easy vector (A1360; Promega) as per the manufacturer’s protocol. The carrier DNA has the same order of base composition, however, the GGG in (*G*_3_ *-N*_5_ *-G*_3_ *-N*_5_ *-G*_3_ *-N*_5_ *-G*_3_) were replaced with ATG. The sequences of the control and carrier DNA are provided below:

The sequence of the Control DNA:

5′-TAATACGACTCACTATAGGGCACCCCTACATCGCAGCGGTCTTTCGGGCTAACGGGATCATGGGACTCAGGGGACAGCCTCAAGCAACATGCCCAGTCCTGACCTTCAATAAGGAAGCAAACTGGGAAGGAGGGTGTCAGGGATAAAGGGGAGTCCTAGTCAAGGTGTCGGATGTCCTAAGACTTATGATCATTTTCTTAGGGTCTAAGGGCTCGAGGGTCTGCGGGTCGGTTTCCTTCTAGAATTAGTATCTTCTATAGTGTCACCTAAAT-3′.

The sequence of the carrier DNA:

5′-TAATACGACTCACTATAGGGCACCCCTACATCGCAGCGGTCTTTCATGCTAACATGATCATATGACTCAATGGACAGCCTCAAGCAACATGCCCAGTCCTGACCTTCAATAAGGAAGCAAACTATGAAGGAATGTGTCAATGATAAAATGGAGTCCTAGTCAAGGTGTCGGATGTCCTAAGACTTATGATCATTTTCTTAATGTCTAAATGCTCGAATGTCTGCATGTCGGTTTCCTTCTAGAATTAGTATCTTCTATAGTGTCACCTAAAT-3′.

### DNA sample preparation for biophysical characterization

Commercially synthesized single-stranded 5′-GGGTCTAAGGGCTCGAGGGTCTGCGGG-3′ (oligo1) and 5′-CCCGCAGACCCTCGAGCCCTTAGACCC-3′ (oligo2) oligodeoxyribonucleotide sequences were purchased from Bioserv Biotechnologies, India. These oligodeoxyribonucleotides were diluted to a final concentration of 40 μM in 50 mM Tris-HCl (pH 7.4) buffer with different KCl concentrations (0.05 M, 0.1 M, 0.15 M, 0.5 M and 1 M) for oligo1 and 0.05 M KCl concentration for oligo2. Subsequently, the DNA samples (oligo1 alone or called DG) and oligos 1 and 2 together (called WC) were heated at 95°C for 5 minutes and cooled down to room temperature for 3 hours. The secondary structural conformational preferences of the DNA oligonucleotides were explored by acquiring the CD spectra immediately after the sample preparation.

### Circular Dichroism (CD) spectroscopy

The CD spectroscopic study was carried out in a JASCO-1500 spectrophotometer at 25°C, and the spectra were processed using spectral manager software (https://jascoinc.com/). The spectral data were collected in triplicate in the wavelength region of 190-320 nm with 1 nm bandwidth in a 1 mm path length cuvette. The baseline correction was done using the Tris-HCl buffer in the presence of an appropriate KCl concentration. Note that only 0.05 M KCl was used for WC, unlike DG. The triplicate average of the CD spectra was considered for the analysis. Further, the thermal melting was carried out for the DG by heating the sample in the range of 15°C to 95°C temperature at 1°C interval and a 30-second incubation time before the acquisition of data at each temperature. The triplicate averaged thermal denaturation curve was used for the analysis.

### Electrophoretic mobility shift assay

Electrophoretic mobility shift assay (EMSA) was carried out using 14% native polyacrylamide gel pretreated with ethidium bromide (EtBr). The native polyacrylamide gel was prepared using 1X TAE buffer (pH 7). Subsequently, 25 μM concentration of the DG sample in 0.05 M Tris-HCl (pH 7.4) buffer with varying concentrations of KCl (0.05 M to 1 M) was mixed with 50% of glycerol and loaded into the well. The electrophoresis was carried out using 1X TAE running buffer at 60 V, 4°C for two hours. After the completion of electrophoresis, the gel was photographed under UV light using SmartView Pro 1100 Imager System, UVCI-1100 from Major science.

### Modeling of DNA conformation

Based on the information obtained from CD and EMSA, the secondary structural preference of DG was modelled using 3D-NuS web server [64]. Pymol 2.1.1 software package (https://pymol.org/2/) was used for the visualization of the generated models.

### Cell culture and transfection of control DNA

HEK293T cells were transduced with a control (CT) or CGGBP1-targeting (KD) shRNA as described elsewhere [5]. These cells were maintained in DMEM (SH30243.01, Cytiva HyClone) supplemented with 10% heat-inactivated fetal bovine serum (FBS) (10270106, Invitrogen) and selected with 0.3 μg/ml of the final concentration of puromycin (CMS8861; HiMedia). For the equality of transfection of the Control DNA, the cloned-in carrier DNA in the same vector was cotransfected in CT and KD using the calcium-phosphate transfection method. 1.25 μg of each of the Control DNA and the carrier DNA (cloned in pGEM-T Easy vector) and 20 μl of 2M CaCl_2_ were mixed. A final volume of 200 μl is attained with sterile autoclaved water. This mix was added dropwise in 200 μl of 2X HEPES buffered saline (51558; Sigma) with gentle agitation on a vortex mixer to form calcium phosphate-DNA precipitate. The precipitate was incubated at room temperature for 20 minutes and added drop-by-drop on approximately 2 × 10^6^ cells per 10 cm dish. On the next day, the cells were processed for *AbC* G4-ChIP.

### *In vitro* immunoprecipitation of the control and the carrier DNA using 1H6

1.25 μg of each of the Control DNA and the carrier DNA were mixed in a 1.5 ml centrifuge tube at room temperature, and the volume was made up to 100 μl using deionized autoclaved water. 22.5 μl of 1 M KCl was added to the final concentration of 150 mM in a final volume of 135 μl. This mix was heated in a dry bath at 95°C for 5 minutes. It was cooled down at room temperature and continued for a further 30 minutes to allow the formation of G4s. Following this incubation, 15 μl of fetal bovine serum (SV30160.03; Cytiva HyClone) was added to the final concentration of 10% v/v. The formed G4s were allowed to be recognized by 1 μl (1 μg) of Anti-G4 1H6 antibody. The mixture was flicked a couple of times and incubated at 4°C for 1 hour. The antibody-G4 interaction was crosslinked using 4 μl of 37% formaldehyde to the final concentration of 1% at room temperature for 10 minutes with a gentle invert-mixing. 15 μl of 1.25 M glycine was added to the final concentration of 125 mM, gently mixed and incubated for 5 minutes to quench the reaction. These antibody-crosslinked G4s were included in the cell lysates for samples CT^*ut*^ and KD^*ut*^ and sonicated, as mentioned in *AbC* G4-ChIP.

For *in vitro* assays described in Supplementary Figure 22, G4s formed on Control DNA *in vitro* were immunoprecipitated using the same protocol as described above. Eluted products were subjected to qPCR assays as described later.

### G4 DNA-IP

Genomic DNA from HEK293T cells was isolated using the phenol-chloroform method stored in 10 mM Tris (pH 8) at −20°C. 1 μg of genomic DNA was denatured at 95°C for 5 minutes and then snap-chilled for the sample G4 DNA-IP^*denat*^. Following this step, the sample was incubated with 10% v/v FBS. At this point 1 μg of genomic DNA, for the sample G4 DNA-IP^*nat*^ also incubated with 10% v/v FBS. The rest of the steps were followed as described for the *in vitro* immunoprecipitation of the Control and carrier DNA.

### *AbC* G4-ChIP

G4s in CT^*nuc*^, KD^*nuc*^, CT^*tr*^, KD^*t*r ^, CT^*ut*^ and KD^*ut*^ were captured using the *AbC* G4-ChIP protocol. The culture medium was removed from 10 cm dishes with or without transfection for each sample. The cells were gently washed with ice-cold 1X Ca^2+^-free PBS (SH30256.01, Cytiva HyClone) twice. After the last wash, the cells were treated with 1 ml of trypsin per dish and incubated at 37°C for 10 minutes. Following the trypsinization, 5 ml of serum-containing DMEM was added to each dish, and the cells were declumped by vigorous pipetting. Then the cells were collected in a clean 50 ml screw-capped centrifuge tube by passing the cell suspension through a 40 μm cell strainer (431750; Corning). The volume was adjusted to a total of 30 ml with serum-free DMEM. The cells were crosslinked with 4 ml of 37% formaldehyde stock solution and incubated for 5 minutes at room temperature to fix DNA-protein interactions. The suspension of cells was quickly quenched with 4 ml of 1.25 M Glycine along with 4 ml of 1 M KCl. The suspension was quickly spun down gently at 1,250 rpm for 5 minutes at room temperature and resuspended in 5 ml of Tris-KCl buffer (50 mM Tris, pH 7.5; 150 mM KCl). The cells were washed once more in the Tris-KCl buffer, spun down at 1,250 rpm for 5 minutes at room temperature, and the supernatant was carefully removed. The cell pellet was resuspended and permeabilized in 15 ml of Tris-KCl buffer supplemented with 0.5% of Triton X-100 for 10 minutes at room temperature with gentle invert mixing. Following the incubation, the suspension was spun down gently at 1,250 rpm for 5 minutes at room temperature and resuspended in 15 ml of Tris-KCl buffer supplemented with 0.01% v/v of Triton X-100 (called TKT buffer) to maintain the permeability of the cell membrane. To the suspension, 10% v/v FBS was added immediately and incubated at room temperature for 30 minutes (for the first 15 minutes with intermittent invert-mixing and then stationary until the end of the 30th minute). The suspension was spun down at 1,250 rpm for 5 minutes at room temperature, and all but 5 ml of the supernatant was gently removed without disturbing the pellet. The pellet was resuspended gently with the help of a pipette to avoid mechanical rupturing of the cells, and the suspension of cells was then transferred into a fresh 15 ml screw-capped tube. This suspension of cells was incubated with anti-G4 1H6 antibodies (Sigma; MABE1126) as 5 μg of antibody/100 μl of cell pellet for two hours at room temperature. Following the incubation, the suspension was spun down at 1,250 rpm for 5 minutes at room temperature, and the pellet was gently washed once with the TKT buffer and resuspended again in the same. The antibody-G4 interactions were then crosslinked with 1% final concentration of formaldehyde and incubated at room temperature for 10 minutes with gentle invert-mixing. 125 mM of the final concentration of glycine was added to quench formaldehyde, and the mix was incubated at room temperature for 5 minutes. The cells were pelleted down at 1,250 rpm for 5 minutes at room temperature and lysed in SDS lysis buffer (50 mM Tris-HCl, pH 8.0; 1% SDS and 25 mM EDTA), supplemented with 1X v/v Halt Protease and Phosphatase inhibitor (87786; Thermo Fisher Scientific), twice the volume of cell pellet. The cells were lysed on ice for 30 minutes with intermittent tapping of the tube. The lysate was equally distributed into 1.5 ml centrifuge tubes (no more than 500 μl/tube) and sonicated in Diagenode Bioruptor sonicator for 20 cycles (with 30 seconds ON - 30 seconds OFF) at constant preset power. The sonicated fraction was quickly pelleted down at 4°C and the supernatant was stored separately at 4°C. To increase the recovery of sonicated fraction, the pellet was resuspended in an equal volume of ChIP dilution buffer (0.01% SDS; 1% Triton X-100; 1.2 mM EDTA; 16.7 mM Tris-HCl, pH 8.0 and 167 mM NaCl) as the volume of sonicated fraction stored earlier. At this stage, the antibody-crosslinked Control DNA (for samples CT^*ut*^ and KD^*ut*^) was included and sonicated for 10 cycles (with 30 seconds ON - 30 seconds OFF) at constant power. The respective sonicated fractions were pooled and centrifuged at 14,000 rpm for 30 minutes at 4°C. Meanwhile, Protein G Sepharose^®^ 4 Fast Flow (GE; 17061801) beads were washed thrice gently with 1X PBS. The beads were gently centrifuged during every wash at 1,000 rpm for 3 minutes to remove the supernatant. The clear sonicated fraction was transferred into a 15 ml tube and diluted with the ChIP dilution buffer to ten times of the volume of SDS lysis buffer used. 10% of this diluted fraction was kept as input. In the rest, 10% v/v slurry of beads was added and tumbled at low rpm for two hours at 4°C. The beads were gently collected by centrifugation at 1,000 rpm for 3 minutes at room temperature and washed with PBST (0.1% Triton X-100 in 1X PBS) five times. The last wash supernatant was removed. Finally, the beads were vigorously vortexed in the presence of an elution buffer (1% SDS; 0.1 M sodium bicarbonate) for 30 minutes. The elution was pooled in 500 μl volume by collecting 250 μl of eluted fraction after 15 minutes and another 250 μl at the end of the 30th minute. The eluted fractions and the inputs were subjected to reverse-crosslinking by adding 20 μl of 5 M NaCl and incubating overnight at 65°C. The reverse-crosslinked DNA was digested with Proteinase K (2 μl of 10 mg/ml stock) in the presence of 10 μl of 0.5 M EDTA and 20 μl of 1 M Tris-HCl (pH 6.8) at 42°C for 1 hour. The DNA was purified using the ethanol precipitation method and dissolved in deionized autoclaved water.

For each sample, eight 10 cm dishes were pooled and subjected to the *AbC* G4-ChIP protocol.

### Library preparation, basecalling, sequence alignment and annotation

The CT^*nuc*^, KD^*nuc*^ and Input^*nuc*^ were sequenced on the IonTorrent S5 sequencer while CT^*tr*^, KD^*tr*^, CT^*ut*^, KD^*ut*^ and respective inputs were sequenced on MinION Mk1B device from the Oxford Nanotechnologies (ONT) sequencing platform. The sequencing libraries were prepared with either Ion Xpress™ Plus Fragment Library Kit (Cat. No. 4471269) or PCR sequencing kit (SQK-PSK004) for the ONT platform as per the manufacturer’s instructions. To generate sufficient sequencing depth, the sequencing library for each sample was pooled from four replicates every time. The samples G4-ChIP, *in vitro* G4 DNA-IPs and the corresponding inputs were sequenced on MinION Mk1B as well. The base-calling of the reads was carried out in real-time. For reads sequenced on MinION, the poor-quality reads were further subjected to basecalling by a locally installed GPU-based *guppy_basecaller*. The sequenced reads were subjected to removal of the sequencing adapter using Porechop using *default* options. The repeat-unmasked hg38 genome (excluding unannotated, mitochondrial chromosomes and chromosome Y) with or without the control DNA was indexed by using *minimap2 -x map-ont* option. The filtered reads were aligned using *minimap2* on the indexed genome, however, the samples, CT^*nuc*^, KD^*nuc*^ and Input^*nuc*^, were aligned on the indexed genome without the control DNA. Only uniquely aligned reads were fetched for further analyses. The sequence data are deposited to NCBI GEO vide ID GSE202456.

### Immunoprecipitation of CGGBP1 of HEK293T cells

One fully confluent 10 cm dish of HEK293T was washed with ice-cold 1X PBS and pelleted at 1,250 rpm in a 1.5 ml microcentrifuge tube at room temperature. The pellet was lysed with RIPA lysis buffer (50 mM Tris, pH 8; 150 mM NaCl; 1% NP-40; 0.5% sodium deoxycholate and 0.1% SDS) added with 1% v/v of Halt Protease inhibitor cocktail at 1:1 (pellet:RIPA lysis buffer) ratio. The lysis was carried out on the ice for 30 minutes with gentle tapping every 10th minute. The lysate was spun down at 14,000 rpm for 30 minutes at 4°C. 0.05% of the clear supernatant was stored as input, while in the rest, 2 μl of anti-CGGBP1 antibody (PA5-57317; Invitrogen) was added and incubated overnight at 4°C. Following the incubation, a 10% v/v slurry of protein A agarose beads was added and incubated at room temperature for two hours. The beads were then washed thrice with RIPA lysis buffer, spun at 1,000 rpm for two minutes, and the supernatant was discarded. The beads were finally washed with ice-cold 1X PBS, spun at 100 rpm for two minutes, and the supernatant was discarded. The beads were then subjected to vigorous vortexing in the presence of elution buffer (0.2 M glycine and 10 mM Tris, pH 8) and eluted by centrifugation at high speed. The eluted CGGBP1 was stored at -20°C until further use.

### Quantitative PCR-stop assay using control DNA and CGGBP1

Quantitative PCR (qPCR) stop assay was performed as described elsewhere [59]. The polymerase employed in our qPCR stop assays was exonuclease and proof-reading activity deficient Klenow fragment (M0212L; New England Biolabs). The Control DNA (Figure 3A) was generated with a T7 sequence (5′-TAATACGACTCACTATAGGG-3′) at the 5′ end and an SP6 sequence (5′-TTCTATAGTGTCACCTAAAT-3′) at the 3′ end. The G-rich strand of the Control DNA was generated using T7 primer and used as a template in qPCR stop assays. 100 ng of double-stranded Control DNA was used as a template per reaction with 250 uM of each dNTP, 10 picomoles of T7 primer, 1X ThermoPol reaction buffer and 1.25 units of Taq DNA Polymerase (M0267L; New England Biolabs) in a 50 μl reaction volume. The following PCR condition was used: (95°C for 5 minutes) X 1 cycle, (95°C for 5 minutes, 55°C for 30 seconds and 72°C for 45 seconds) X 25 cycles and (72°C for 5 minutes) X 1 cycle. The single-stranded DNA generated using this method was purified using PCR purification kit (A9285; Promega) and quantified using Nanodrop. 200 ng of single-stranded G-rich DNA was used in qPCR stop assay. The reaction mix comprised of 250 uM of each dNTP, 10 picomoles of SP6 primer, 1X reaction buffer and 1.5 units of Klenow (M0212L; NEB). The SP6 primer (5′-ATTTAGGTGACACTATAGAA-3′) at the 3′ terminus of the G-rich strand was extended such that the amount and the length of the primer extension products obtained were inversely proportional to the polymerase stop exerted by G4s. As described in various experiments, the purified single-stranded G-rich DNA was incubated with 150 mM KCl (P9541; SIGMA) or 150 mM LiCl (L9650; SIGMA) at 95°C for 5 minutes, followed by at room temperature for 30 minutes before incubation with Klenow for polymerase activity and primer extension. In experiments where the effects of CGGBP1 were studied, the single-stranded G-rich DNA was preincubated with rCGGBP1, or immunoprecipitated CGGBP1 or isotype-control IgG immunoprecipitates at room temperature for 30 minutes followed by incubations with KCl or LiCl and the primer extension steps. The single-stranded primer extension assay products were quantified using a primer pair (5′-CAGCCTCAAGCAACATGCCCAGTC-3′ and 5′-AGTTTGCTTCCTTATTGAAGG-3′), located downstream from the SP6 site on the Control DNA closer to the T7 site. The specific amplification from the primer pair was established by resolving the PCR products on agarose gel and visualization using Ethidium bromide staining. qPCRs were performed on a HiMedia Insta Q96 real-time PCR machine using the iTaq Universal SYBR Green Supermix (1725125, Bio-Rad) as the fluorophore for PCR product real-time quantification. Each qPCR was performed in triplicates or more replicates, as indicated in the figures. Melt curves were obtained to ascertain PCR specificity in each run. The Ct values were obtained for amplification curves with comparable efficiencies as early as possible at the onset of the logarithmic phase of the PCR. The Ct values were exported in text files and analyzed using the double delta Ct method in LibreOffice spreadsheets. Data were plotted using GraphPad PRISM 9. Outliers were excluded from the analyses. The statistical significance of differences was calculated using indicated *t*-tests inbuilt in PRISM 9.

### Generation of correlation matrices

The human genome was binned into 0.2 kb bins. Coverage of the uniquely aligned reads on those genomic bins was calculated using *bedtools coverage* with the option *-counts*. Any genomic bins with read coverage of less than 3 and more than 100 were excluded. The bins satisfying this criterion were used to fetch the reads from all the samples and the inputs. The reads were then converted into *bam* format using *bedtools bedtobam* function. The bam files were then indexed using *samtools* and converted to *bigWig* signal using *deepTools bamCoverage* with a bin size of 200. These *bigWig* signals were used to compute the correlation using *deepTools multiBigwigSummary*. The heatmaps of correlation matrices were generated using *deepTools plotCorrelation* with the options *-c spearman -p heatmap --skipZeros --removeOutliers*.

### Principal component analysis (PCA)

Principal component analysis was performed by pooling the input-normalized signals for CT, KD and their respective inputs. PC scores were calculated from the coefficients of the linear combinations of the variables (CT, KD, CT-Input and KD-Input). The loadings vectors were plotted as correlations between the variables and the principal components. The loadings and PC scores were plotted in as a biplot in GraphPad PRISM 9.

### G/C-skew calculation

The human genome hg38 was binned in 100bp regions using *bedtools makewindows* and fasta sequences were fetched using *bedtools getfasta* from the unmasked hg38. For every bin, the presence of “G”s and “C”s were calculated separately using *emboss fuzznuc*. Any corresponding bin having a *zero* count was eliminated from the G/C-skew calculation. Once all the bins with “G” and “C” counts were available, G/C-skew was calculated using the formula ((G−C)/(G+C)) for each 100 bp genomic bin. This information was used to generate bigWig signal using UCSC *bedGraphToBigWig* for generating heatmap.

### Discovery of differentially captured regions (DCRs)

The coverage of uniquely aligned sequenced reads was computed for all *AbC* G4-ChIP samples in the 0.2 kb genomic bins. The coverage counts were normalized using sequencing depth in the corresponding CT or KD samples. Those 0.2 kb genomic bins were chosen wherein the raw coverage counts follow the criterion of ≥3 and ≤100 for all three sets of *AbC* G4-ChIP samples. Then the corrected coverage counts were used to calculate the “diff/sum” ratios ((KD−CT)/(KD+CT)) for each pair of the *AbC* G4-ChIP samples. The same exercise was performed for the inputs, however, no limit to coverage counts was applied. Thus, the diff/sum ratios for the three *AbC* G4-ChIP samples and three inputs were calculated for each bin individually, and a separate one-tailed *t*-test was applied for each bin with *n* = 3 for CT as well as KD. The 0.2 kb genomic bins with *p*-value < 0.01 (true discoveries established through *q*-value < 0.01) were selected and denoted as differentially captured regions (DCRs).

### GGG/CCC weighted content calculation

GGG/CCC weighted content was calculated on all peak datasets. Fasta sequences for the peaks were fetched from the repeat-unmasked hg38 followed by “GGG” and “CCC” patterns were searched using *emboss fuzznuc* with every 1-base succession for each peak. Any peaks with zero count of either “GGG” or “CCC” patterns were discarded for GGG/CCC weighted content calculation. The weighted GGG/CCC content of a peak was calculated by taking the product of the sum of “GGG” and “CCC” counts and the coverage of reads on the given peak. The GGG/CCC weighted content was plotted using GraphPad PRISM 9.

### Peak calling and annotation

Broad peaks were called on the datasets against the corresponding inputs as background noise using the *MACS2 callpeak* function. For all *AbC* G4-ChIP samples and G4 DNA-IP^*denat*^, a bandwidth of 3000 (*--bw 3000*) was used to call the broad peaks with the options: *--nolambda --broad --broad-cutoff 0.01*. However, for G4-ChIP and G4 DNA-IP^*nat*^ samples, broad peaks were called at the default bandwidth value of 300. The details of the discovered peaks are listed in Supplementary Table 2. The annotation of the peaks (for samples CT^*nuc*^, G4 DNA-IPs and G4-ChIP) based on the genomic landmarks was performed using *annotatePeak* function from the R package *ChIPseeker* [65, 66]. The distance from peak to the TSS was defined from −3 kb to +3 kb and other transcript-related features (from the bioconductor package *TxDb.Hsapiens.UCSC.hg38.knownGene*) were defined using the *TxDb* object. Various genomic features for the peaks were visualized using *plotAnnoBar*. Overlaps of peaks (at least 70%) from different datasets were generated as a Venn diagram by using *vennplot* function.

### Jaccard index calculation

Jaccard indices on the peaks were calculated using the *bedtools jaccard* option regardless of strandedness. The indices between samples are mentioned in Supplementary Figure 8.

### Data availability statement

The data that support the findings of this study are openly available in NCBI GEO with reference ID GSE202456.

## SUPPLEMENTARY MATERIALS




