# Supplementary material for: G-quadruplex landscape and its regulation revealed by a new antibody capture method
Source: Oncotarget. 2024 Mar 14;15:175–98. doi: 10.18632/oncotarget.28564 (PMC10939474; doi:10.18632/oncotarget.28564)
Supplement: Supplementary file 1 [file oncotarget-15-28564-s001.pdf]

G-quadruplex landscape and its regulation revealed by a new antibody capture method

SUPPLEMENTARY MATERIALS

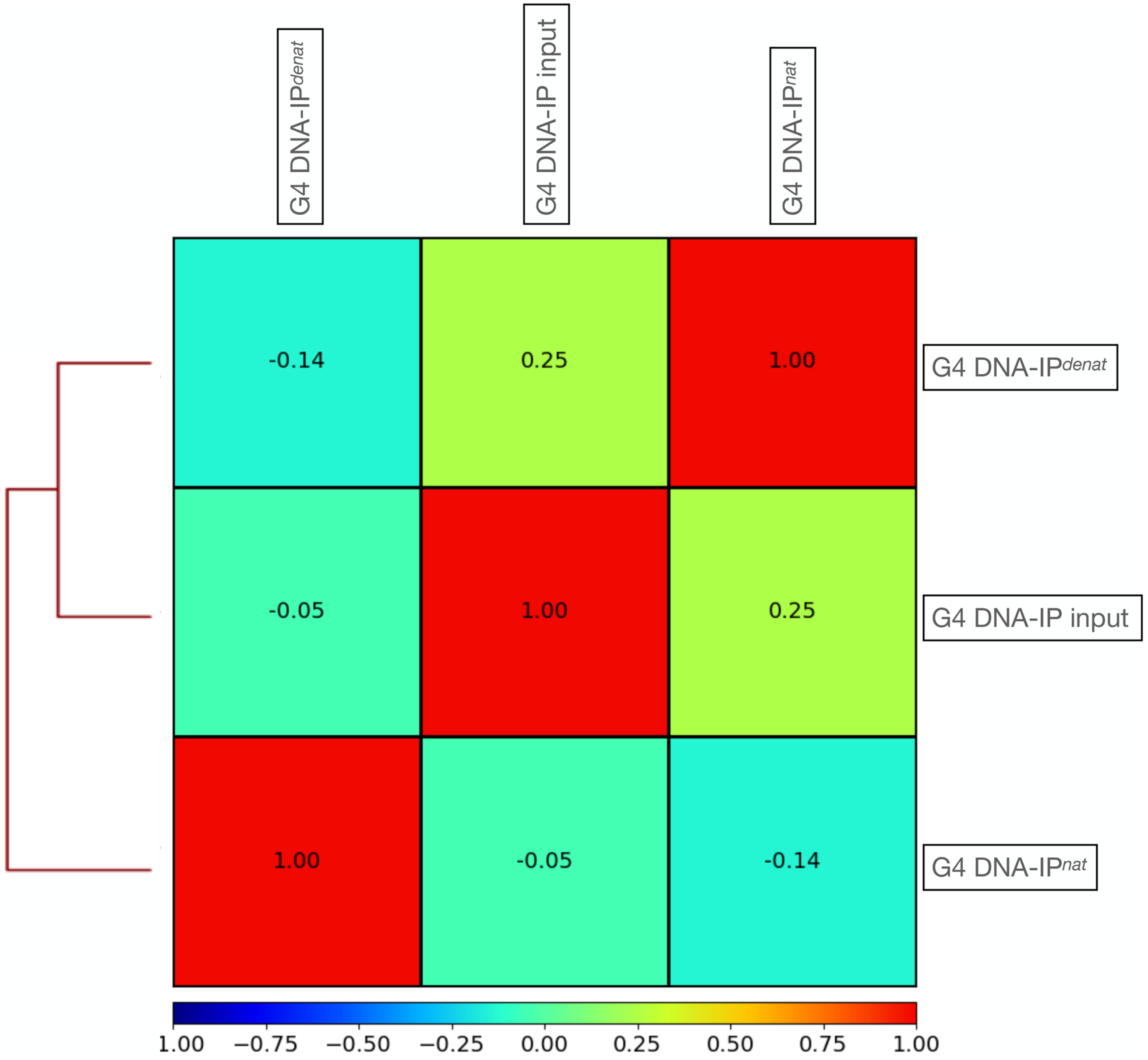

Supplementary Figure 1: Spearman's correlation matrix and clustering of G4 profile for *in vitro* G4 DNA-IPs. Genome-wide comparison of signals in G4 DNA-IP^{nat} and G4 DNA-IP^{denat} against a common input shows that the two IPs differ from the input as well as from each other. Thus, the G4 profiles in G4 DNA-IP^{nat} and G4 DNA-IP^{denat} are very different. However, the slight similarity between G4 DNA-IP^{denat} and DNA-IP input is in sharp contrast to the dissimilarity between G4 DNA-IP^{nat} and G4 DNA-IP^{denat} or DNA-IP input. This suggests the widespread G4 potential of the genome captured in G4 DNA-IP^{denat} and a much more restrictive G4 capture in G4 DNA-IP^{nat}. The signals in the samples were calculated on 0.2 kb bins genome-wide. The numbers indicate Spearman's correlation coefficients for pairs of samples.

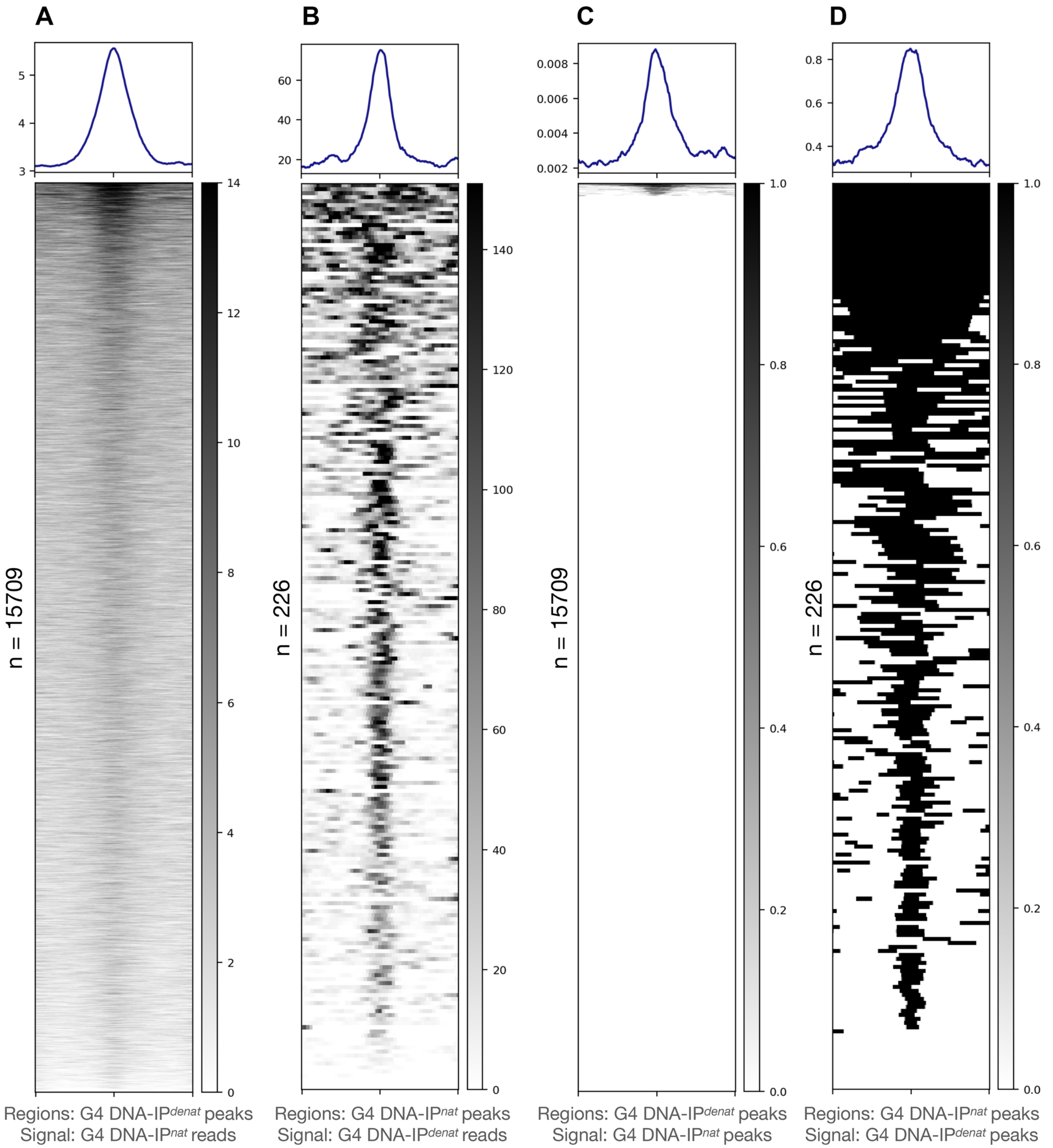

Supplementary Figure 2: Comparison between G4 DNA-IP*nat* and G4 DNA-IP*denat* signals and peaks genome-wide. The G4 signal from G4 DNA-IP*nat* was poor at G4 DNA-IP*denat* peaks (**A**), whereas the G4 DNA-IP*denat* signals were highly enriched at G4 DNA-IP***nat*** peaks (**B**) suggesting that the G4 DNA-IP*nat* pull-down is a subset of G4 DNA-IP*denat* pull-down. A similar pattern was observed more emphatically at the peak level. The small number of G4 DNA-IP*nat* peaks were almost entirely present as a subset of the G4 DNA-IP*denat* peaks (**C**), whereas only a minor fraction of the G4 DNA-IP*denat* peaks overlapped with G4 DNA-IP*nat* peaks (**D**). The signals were plotted on 5 kb flanks from the centers of the peaks. Numbers indicate peaks identified in the respective sample, and the Y-axes of the plots have signals plotted as mean ± SEM.

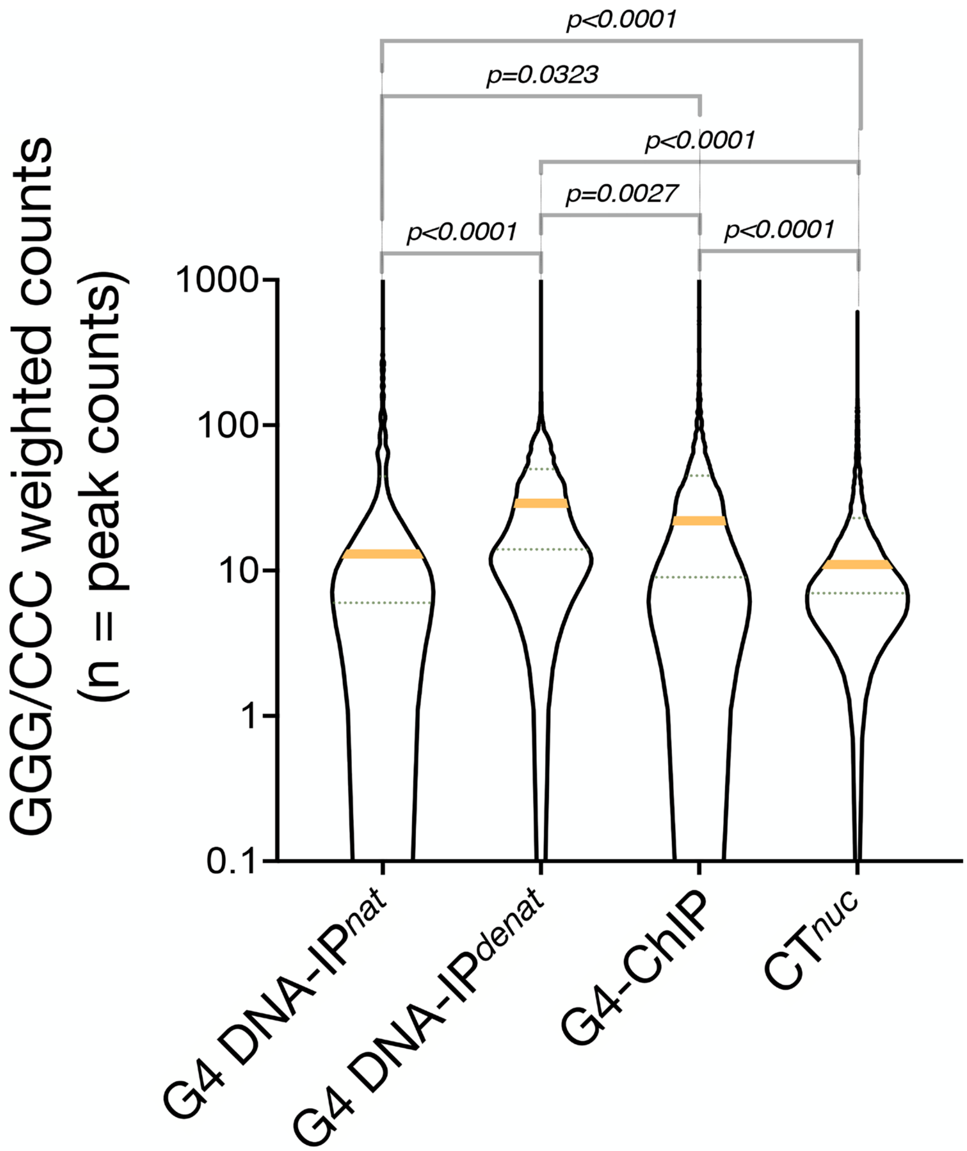

Supplementary Figure 3: Violin plots of read representation-weighted GGG/CCC counts in peaks (product of GGG/CCC count and the number of reads mapping to each peak). The Y-axis shows the median and IQR on a log10 scale. Remarkably, CTnuc has the lowest GGG/CCC representation in peaks. Peak counts and properties are presented in Supplementary Table 2.

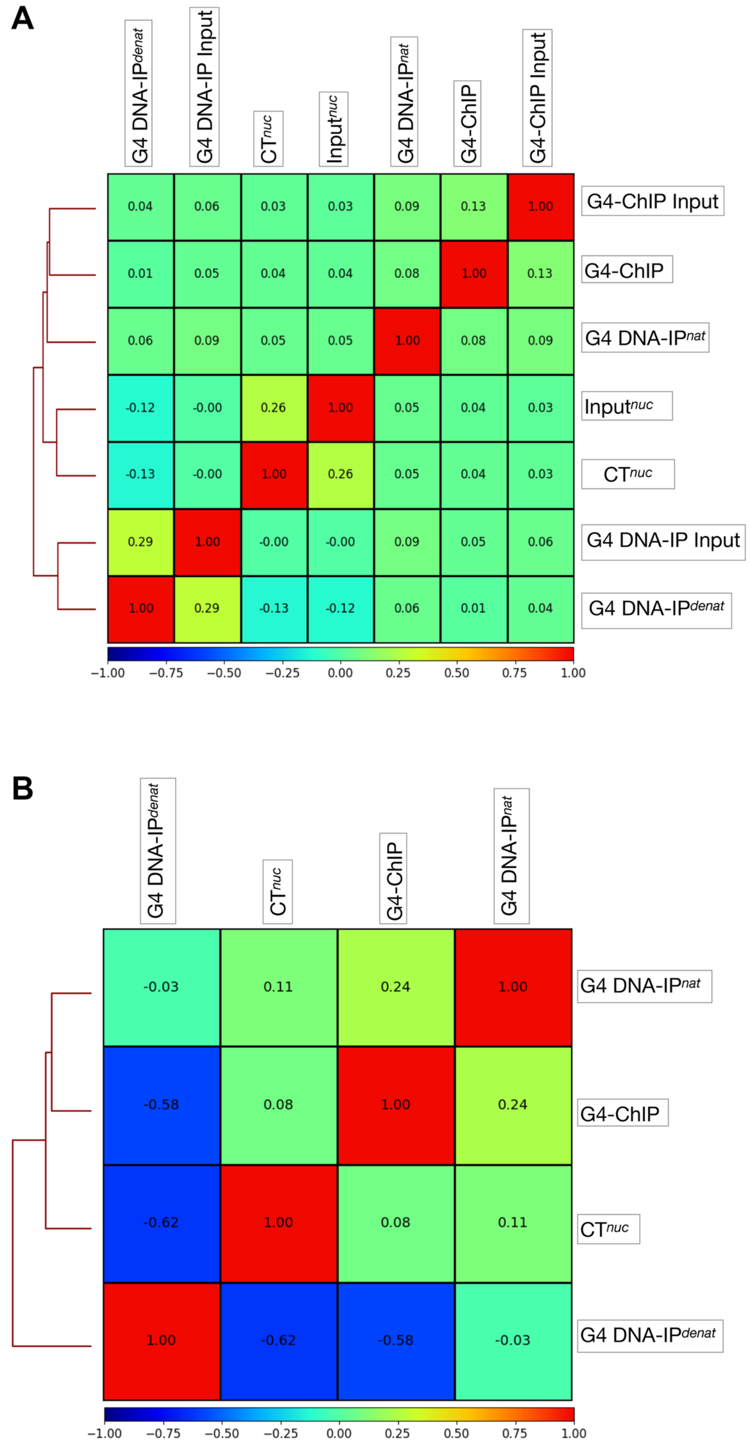

Supplementary Figure 4: Correlation matrix and clustering for the G4 landscapes reported by various methods at signal and peak levels. (**A**) The absolutely DNA-IPs (G4 DNA-IP^{nat} and G4 DNA-IP^{denat}) show similarities with the conventional G4-ChIP, whereas the *AbC* G4-ChIP eliminates this similarity. Notably, all ChIP samples cluster with the respective inputs except for the G4 DNA-IP^{nat,} which co-clusters with the G4-ChIP. (**B**) The peaks in G4 DNA-IP^{nat} correlate with the peaks from G4-ChIP, suggesting that the spontaneously forming G4s in G4 DNA-IP^{nat} are also captured in G4-ChIP. This is unlike is CT^{nuc,} which shows a poorer similarity with G4 DNA-IP^{nat}. G4 DNA-IP^{denat} did not show similarity with G4-ChIP or CT^{nuc}. The numbers in the insets are Spearman's coefficients.

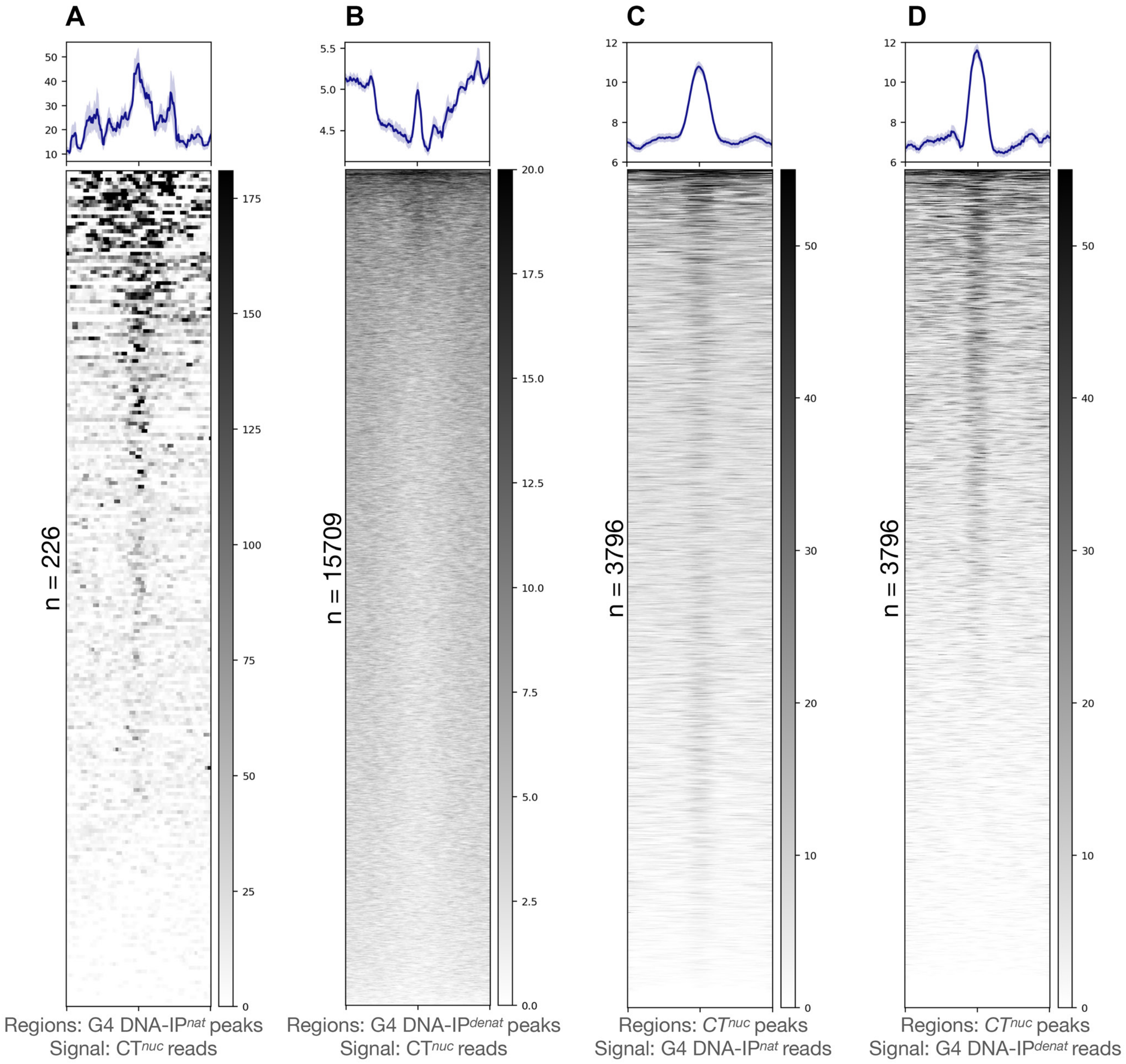

Supplementary Figure 5: Comparison of G4 profiles of CT*nuc* with G4 DNA-IP*nat* and G4 DNA-IP*denat*. (**A**) G4 DNA-IP*nat* peaks show a clear central enrichment of CT*nuc* signals. (**B**) Only a very small fraction of G4 DNA-IP*denat* peaks show enrichment in CT*nuc*. Most of the G4 DNA-IP*denat* peaks are selectively devoid of CT*nuc* signals at the peak centres. (**C**, **D**) In agreement with the other observations described so far, a larger fraction of CT*nuc* peaks contain G4 DNA-IP*nat* signals (C), whereas a much smaller fraction of CT*nuc* peaks contain G4 DNA-IP*denat* signals (D). The signals were plotted on the 5 kb flanks from the centers of peaks. Numbers indicate the peaks identified in the respective sample, and the Y-axes of the plots have signals plotted as mean ± SEM.

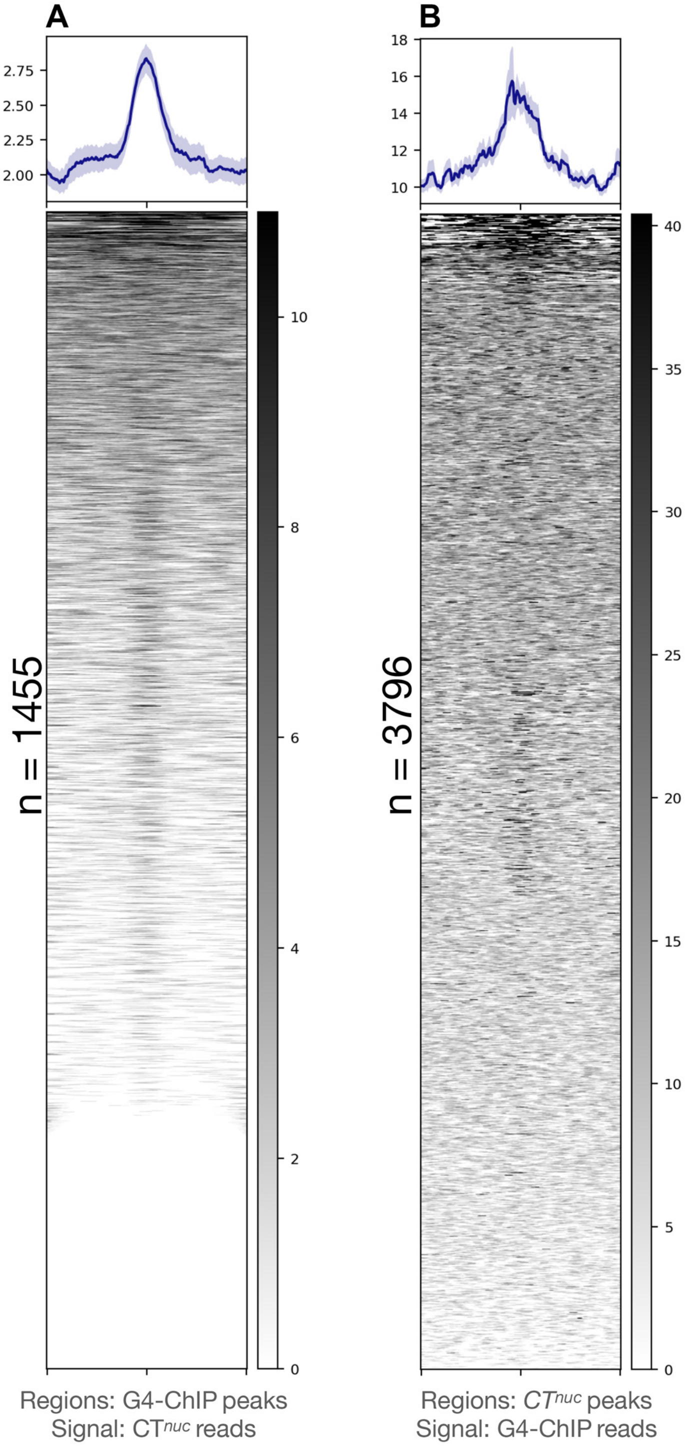

Supplementary Figure 6: A comparison of G4-ChIP and CT*nuc*. (**A**, **B**) At CT*nuc* peaks, there is a weaker enrichment of G4-ChIP signals as compared to the G4-ChIP peaks (A), which show a much stronger signal with a high background of CT*nuc* signals. As expected, a subset of CT*nuc* peaks showed no signal from G4-ChIP, whereas all G4-ChIP peaks showed CT*nuc* signals suggesting that the CT*nuc* G4 profile is a superset of the G4-ChIP profile. The signals were plotted on the 5 kb flanks from the centers of peaks. Numbers indicate the peaks identified in the respective sample, and the Y-axes of the plots have signals plotted as mean ± SEM.

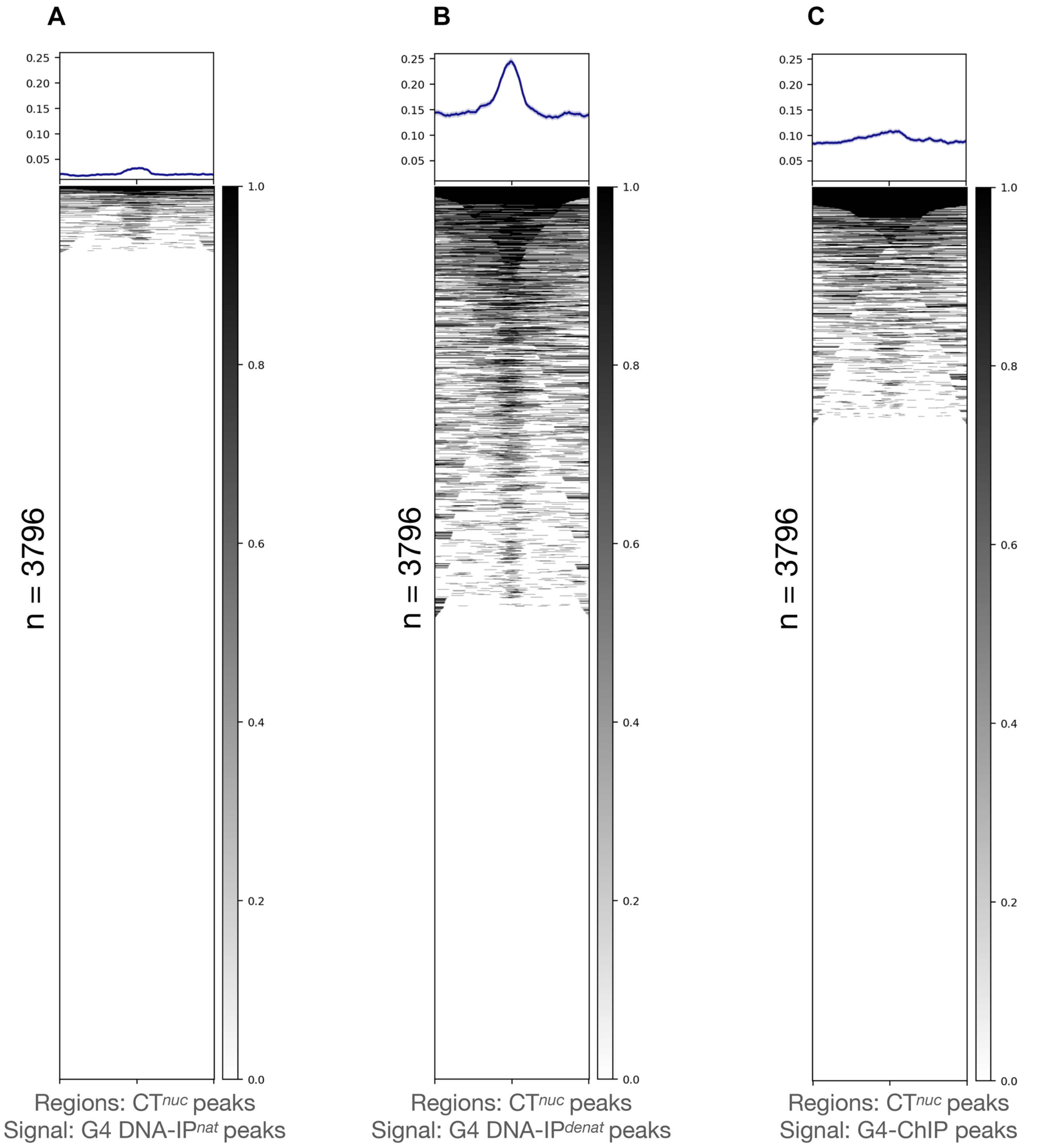

Supplementary Figure 7: A comparison of signals from peaks of DNA-IPs and G4-ChIP on CT^{nuc} peaks. (**A**, **B**) A very small population of CT^{nuc} peaks contain signals from G4 DNA-IP^{denat} peaks with no clear enrichment at the CT^{nuc} peak centers (A) while about half of the CT^{nuc} peaks contained signals with a lot of background from G4 DNA-IP^{denat} peaks (B) suggesting that peaks identified in CT^{nuc} (Supplementary Table 2) is a superset of G4 DNA-IP^{denat} peaks. (**C**) The peak signals from G4-ChIP peaks showed a weaker presence on the CT^{nuc} peaks but with a similar background signal as shown by G4 DNA-IP^{denat} peaks. The signals were plotted on the 5 kb flanks from the centers of peaks. Numbers indicate the peaks identified in the respective sample and the Y-axes of the plots have signals plotted as mean ± SEM.

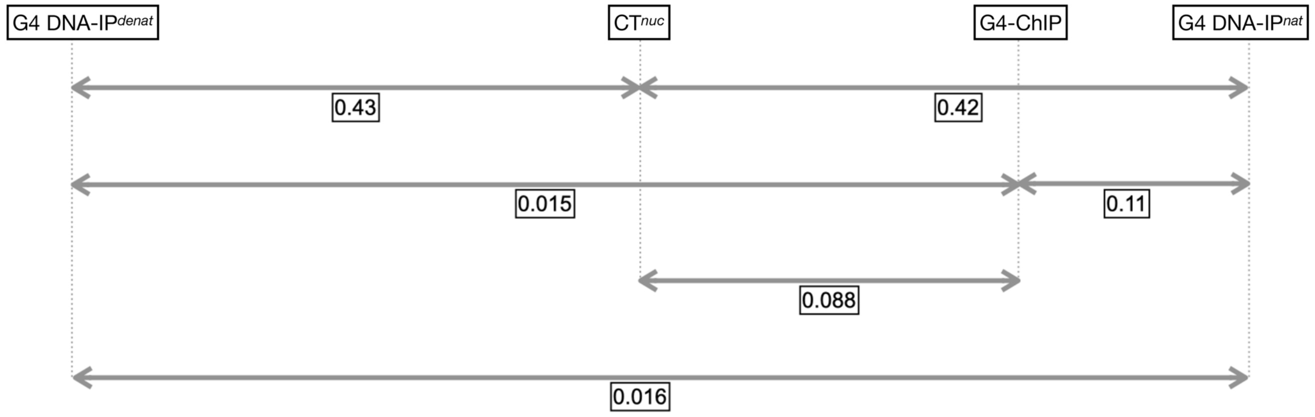

Supplementary Figure 8: Jaccard indices of G4 peaks identified in CT^{nuc}, G4-ChIP, G4 DNA-IP^{denat} and G4 DNA-IP^{nat}. The numbers indicate the proximity of occurrences of G4 peaks genome-wide. The G4 DNA-IP^{denat} represents a very different profile of G4 peaks than in the rest of the samples. The profile of the G4 peaks in G4-ChIP showed a close resemblance to the G4 DNA-IP^{denat} and CT^{nuc}. The lines with the bidirectional arrows indicate the pairs of samples within which the Jaccard indices were calculated. The numbers on the bottom of the lines are not on the scale.

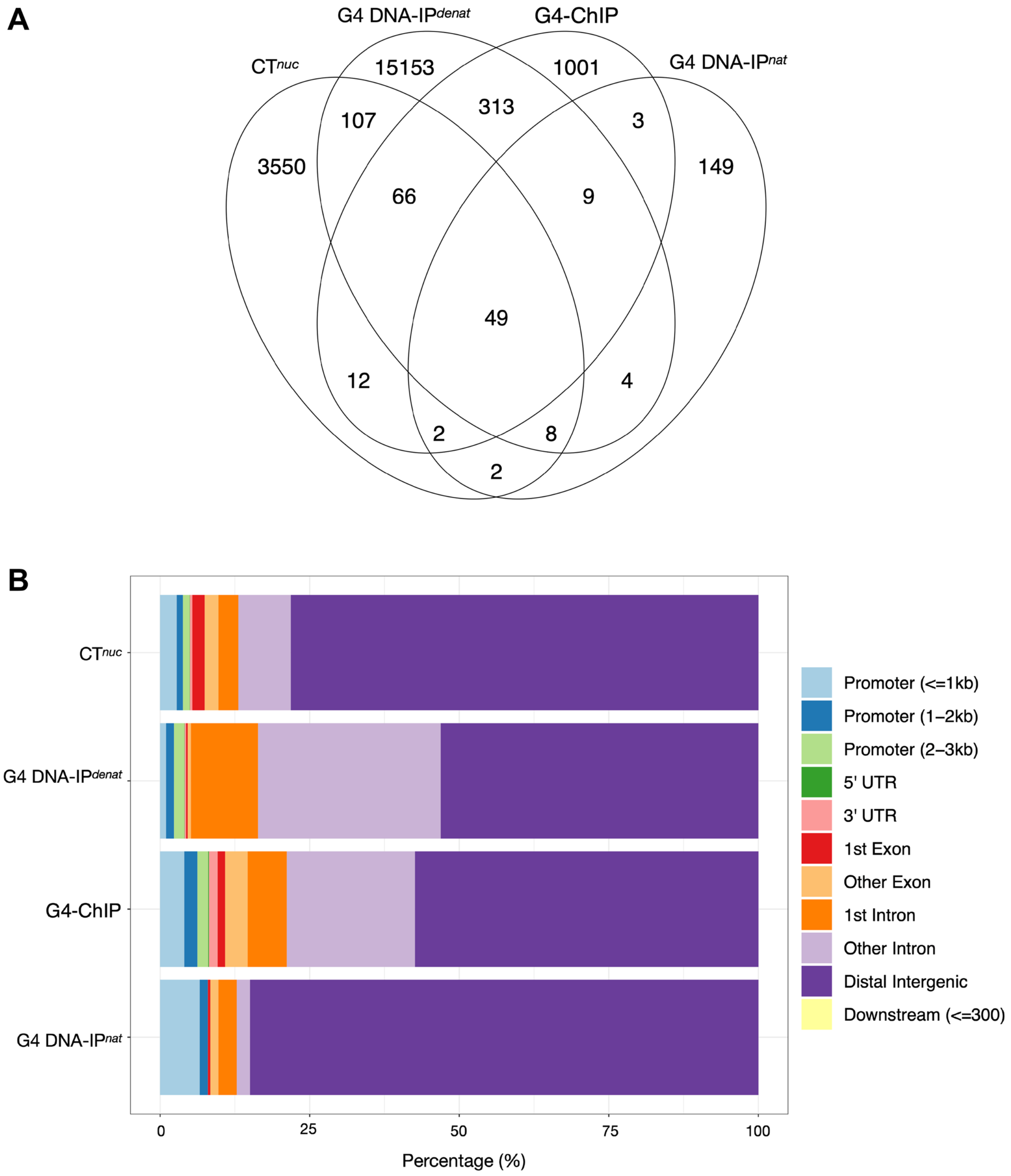

Supplementary Figure 9: Annotation of peaks identified in CT*nuc*, G4 DNA-IPs and G4-ChIP. (**A**) A Venn diagram representation of overlap (≥70% of peak lengths) between peaks from different datasets shows that CT*nuc* is the most rich in unique and non-overlapping peaks. Considering the large number of peaks identified in G4 DNA-IP*denat*, it is striking that the *AbC* G4-ChIP sample, CT*nuc*, has a unique to shared ratio of 3550/230, whereas the G4-ChIP sample has a unique to shared ratio of 1001/437. (**B**) Majority of the peaks identified in these four samples fall in the intronic and intergenic regions. Only a small fraction of peaks fall in the promoter regions. The similarity in distributions of peaks with distal intergenic regions and introns between G4-ChIP and G4 DNA-IP*denat* shows again that G4-ChIP captures G4s which are expected to be found only *in vitro* under conditions which facilitate DNA strand separation.

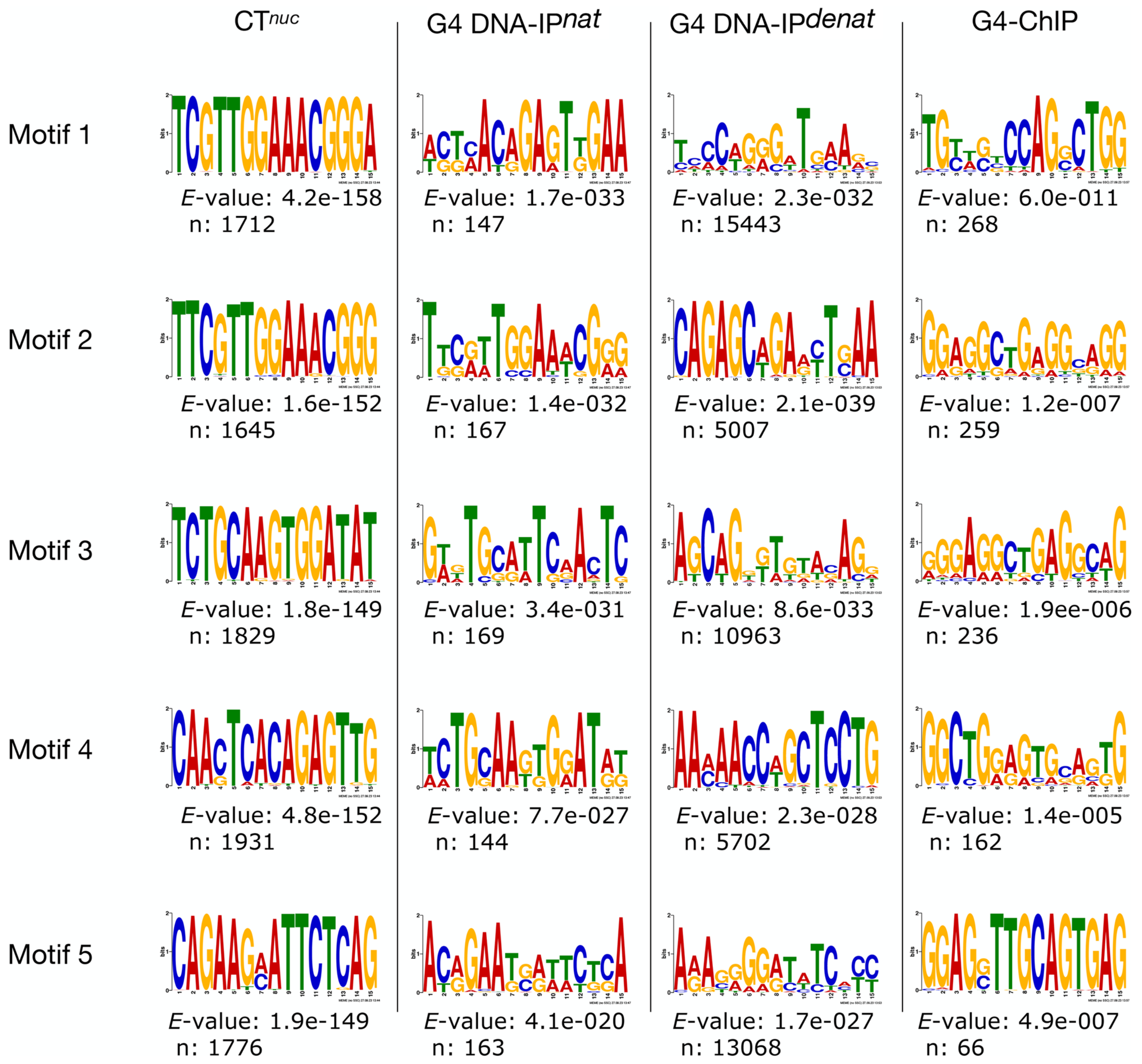

Supplementary Figure 10: Motif discovery in peaks derived from G4 DNA-IP^{nat}, G4 DNA-IP^{denat}, G4-ChIP and CT^{nuc} using MEME. Multiple 15 bps long DNA sequence motifs were discovered in all the peak sets. However, the G-triplets, an expected feature of G4-forming sequences, were discovered with high significance only in the *AbC* G4-ChIP sample CT^{nuc} (Motif 1 and Motif 2 with highest significance in the sample CT^{nuc}). Interestingly, these two highest scoring motifs in CT^{nuc} were near identical and showed no wobble at the G-triplet locations. The other possibilities of G-triplets in motifs discovered in G4-ChIP showed wobble and low probability. The *E*-values and number of motifs discovered (n count) are indicated under each motif diagram.

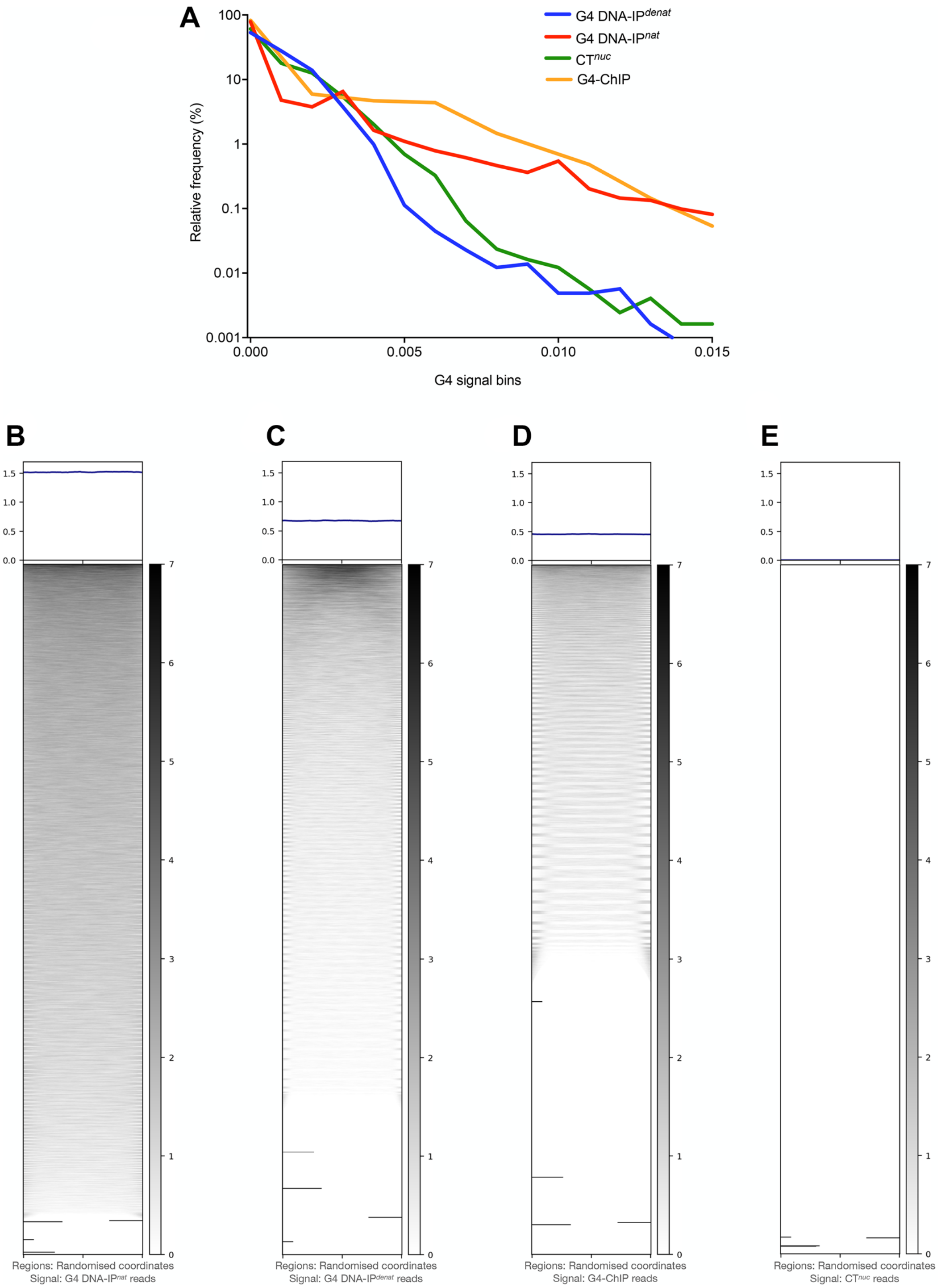

Supplementary Figure 11: A comparison of signals from DNA-IPs, CT^{nuc} and G4-ChIP on randomly drawn genomic regions. (**A**) Using 123190 (summation of 10 rounds of randomization of 0.2 kb genomic bins of 12319 regions, as in DCRs, independently from the hg38) randomly selected regions genome-wide, the G4 signals at random regions were calculated for the four samples as indicated. The CT^{nuc}, in which *AbC* G4-ChIP was applied, showed a very restricted low level detection of G4 signals at these random regions, similar to the G4 DNA-IP^{denat}. The G4-ChIP and G4 DNA-IP^{denat}, on the other hand, showed a relatively high prevalence of G4 capture at random regions. These comparisons showed that the discrete and specific G4s are detected by the application of the *AbC* G4-ChIP protocol, and it minimizes *in vitro* G4s captured at random regions as artifacts. (**B**–**E**) These randomly drawn 123190 genomic regions contain no or poor signal from G4 DNA-IP^{nat} (B), G4 DNA-IP^{denat} (C), G4-ChIP (D) and CT^{nuc} (E), respectively. The signals were plotted on the 5 kb flanks from the centers of the randomized regions. The Y-axes of the plots have signals plotted as mean ± SEM.

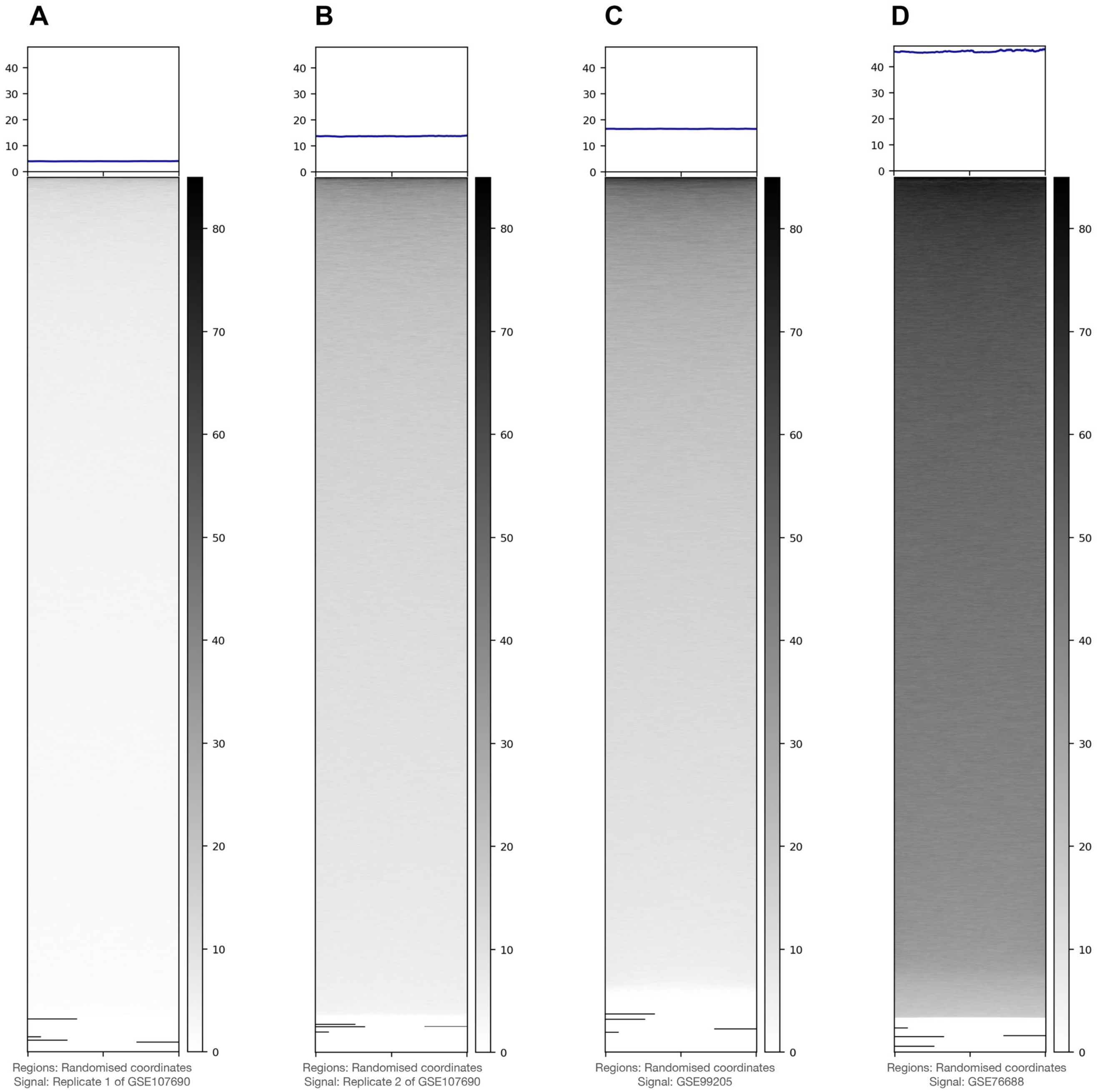

Supplementary Figure 12: A comparison of publicly available G4-ChIP signals on genome-wide randomized regions. (**A**–**D**) Publicly available previously reported G4-ChIP signals (indicated by the GSE datasets mentioned at the bottom of the heatmaps) were plotted on the 5 kb flanks from the centers of genome-wide randomized regions (as in Supplementary Figure 9). Overall, these regions carry a higher background from the reported G4-ChIP signals as compared to G4 DNA-IPs, G4-ChIP and CT^{nuc}. This indicates that antibody 1H6 captures a very restrictive set of G4s. The Y-axes of the plots have signals plotted as mean ± SEM.

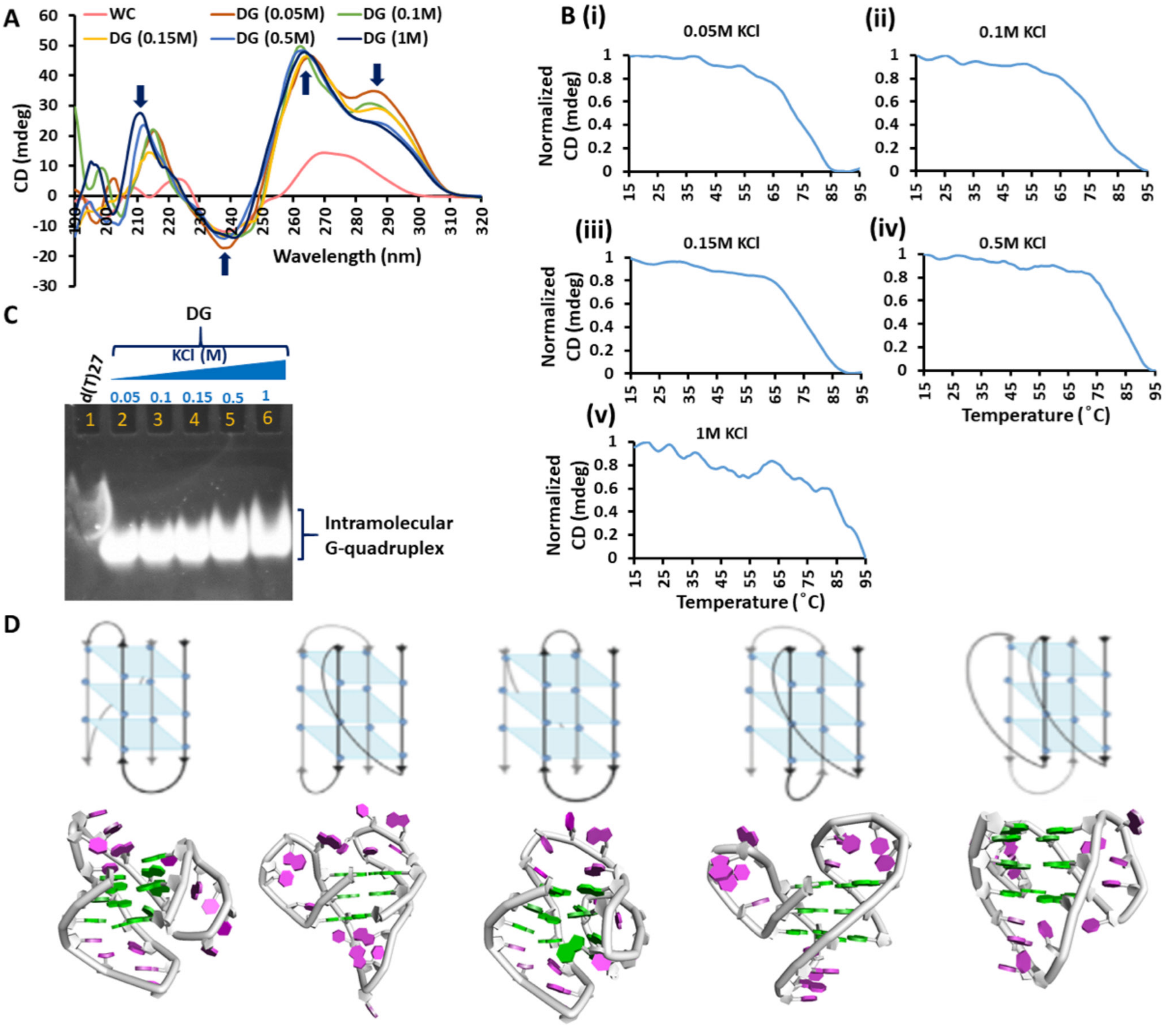

Supplementary Figure 13: CD wavelength scan, CD thermal melt experiment and EMSA showing the preference for intramolecular hybrid G4 by the Control DNA unit sequence (5′-GGGTCTAAGGGCTCGAGGGTCTGCGGG-3′). (**A**) CD spectra showing the preference for the hybrid G4 conformation by the DG in the presence of 50 mM Tris-HCl (pH 7.4) buffer irrespective of different KCl concentrations (0.05 M, 0.1 M, 0.15 M, 0.5 M and 1 M). Note the absence of such a conformational preference by the corresponding WC duplex (WC, colored in peach). (**B**) The hypochromic thermal melting pattern seen for the DG at different KCl concentrations (**i**) 0.05 M, (**ii**) 0.1 M, (**iii**) 0.15 M, (**iv**) 0.5 M and (**v**) 1 M indicates the stable G4 formation. Note that the CD thermal melting was measured at 260 nm. (**C**) EMSA corresponding to the DG indicates the intramolecular G4 formation at different KCl concentrations as it moves faster (Lanes 2-6) than the control $d(T)_{27}$ (Lane 1). (**D**) Possible intramolecular hybrid G4 conformation preferred by the DG, which is modeled using the 3D-NuS web server. The guanines of the G-quartet are colored green, and the loop residues are shown in pink.

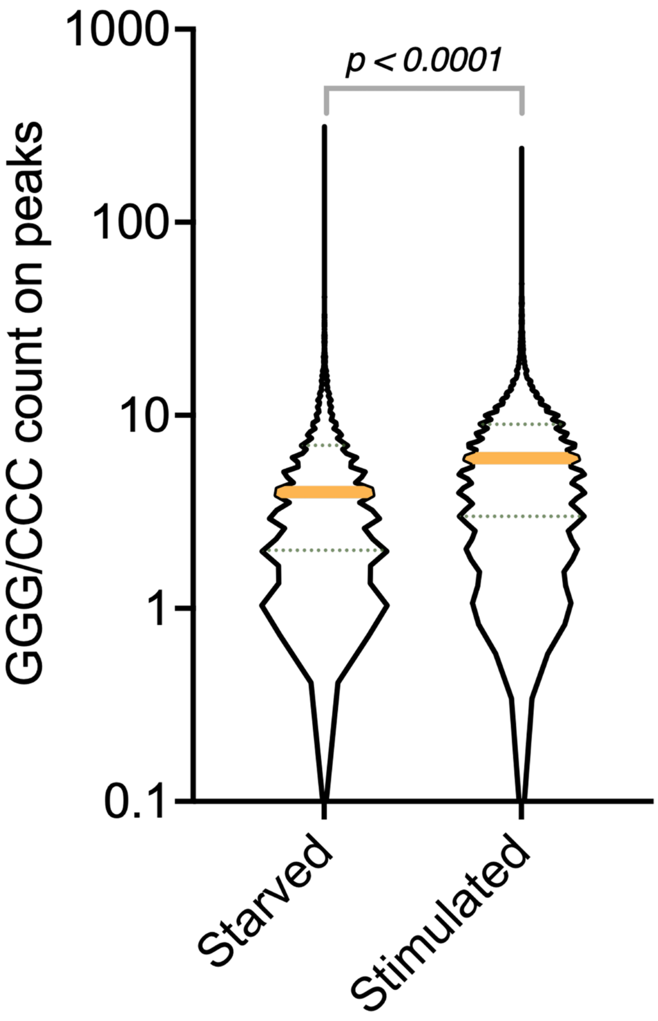

Supplementary Figure 14: GGG/CCC content on CGGBP1 peak datasets (GSE53571). GGG/CCC content calculated in two different CGGBP1 peak datasets (growth-stimulated and starved) shows that the CGGBP1 occupancy is facilitated in genomic regions with higher GGG/CCC content upon growth stimulation. The difference in the CGGBP1 occupancy in these two datasets is denoted by a strong *p-value*. The orange lines indicate the median values presented by the Y-axis on a log10 scale.

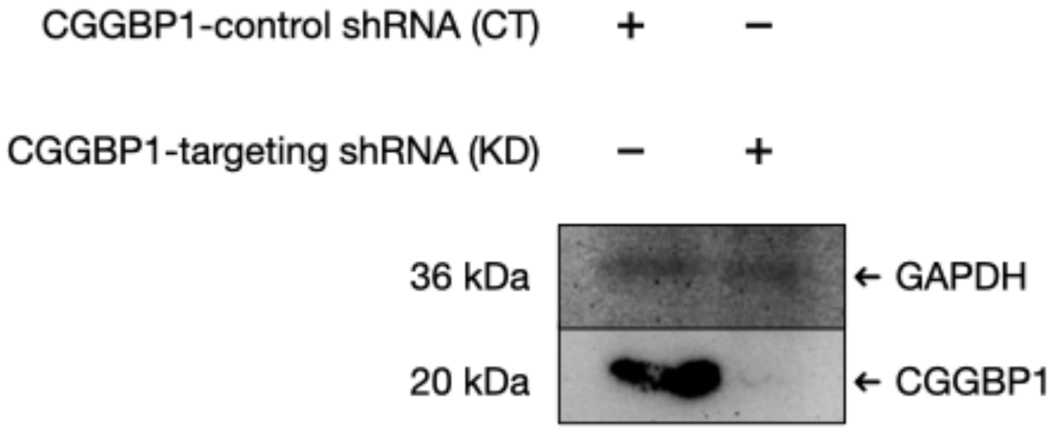

Supplementary Figure 15: Western blot showing the knockdown of endogenous levels of CGGBP1. The levels of CGGBP1 and GAPDH are shown in the lower and upper panels, respectively. The level of knockdown of CGGBP1 is normalized to the levels of GAPDH. Approximately 99% knockdown of endogenous levels of CGGBP1 was observed in KD.

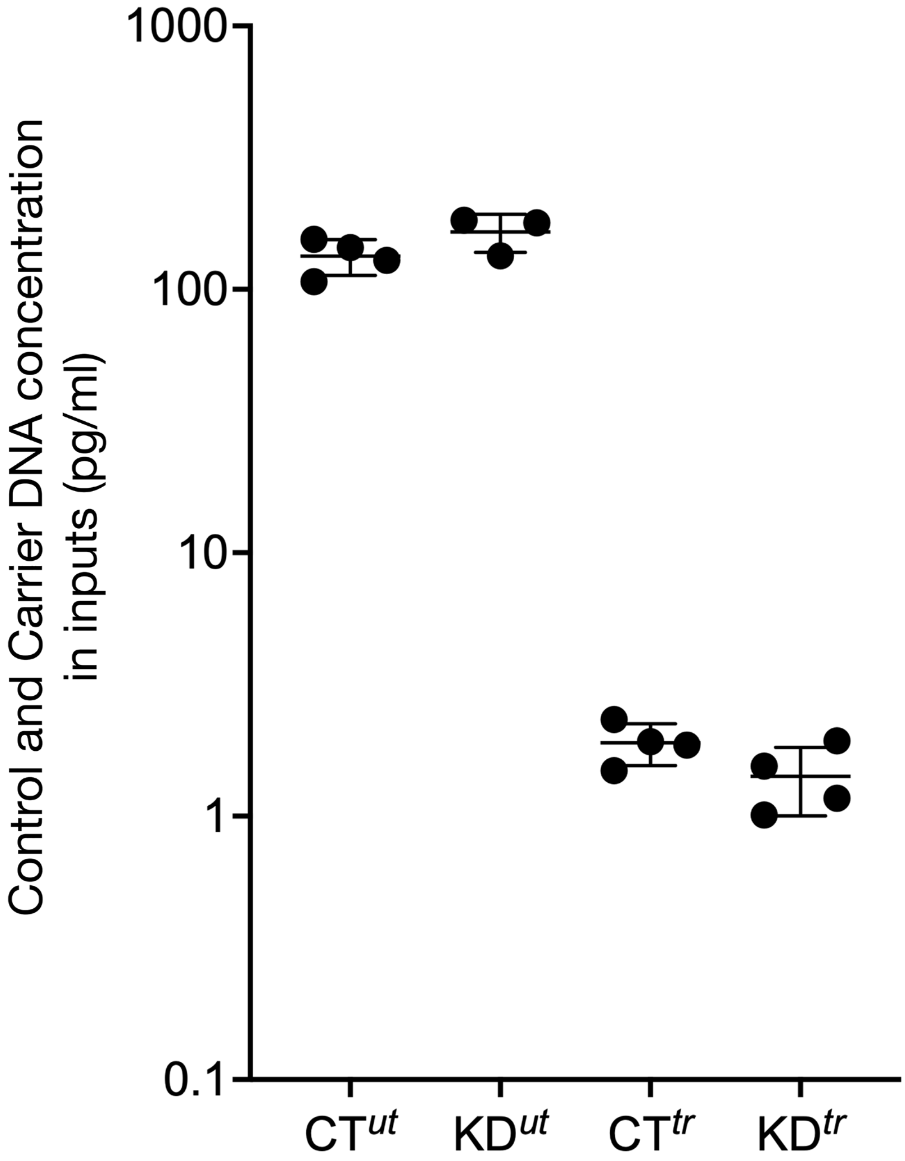

Supplementary Figure 16: Recovery of control and carrier DNA in the inputs of CT^{ut}, KD^{ut}, CT^{tr} and KD^{tr}. The qPCR quantification confirms the bias-free presence of the control and control DNA between CT and KD as quantified in their input samples. Notably, the *in vitro* addition of the control and carrier DNA in the untransfected samples yielded a higher amount of the control DNA than that can be quantified in the transfected samples. The Y-axis represents the concentration of the control and control DNA on a log10 scale.

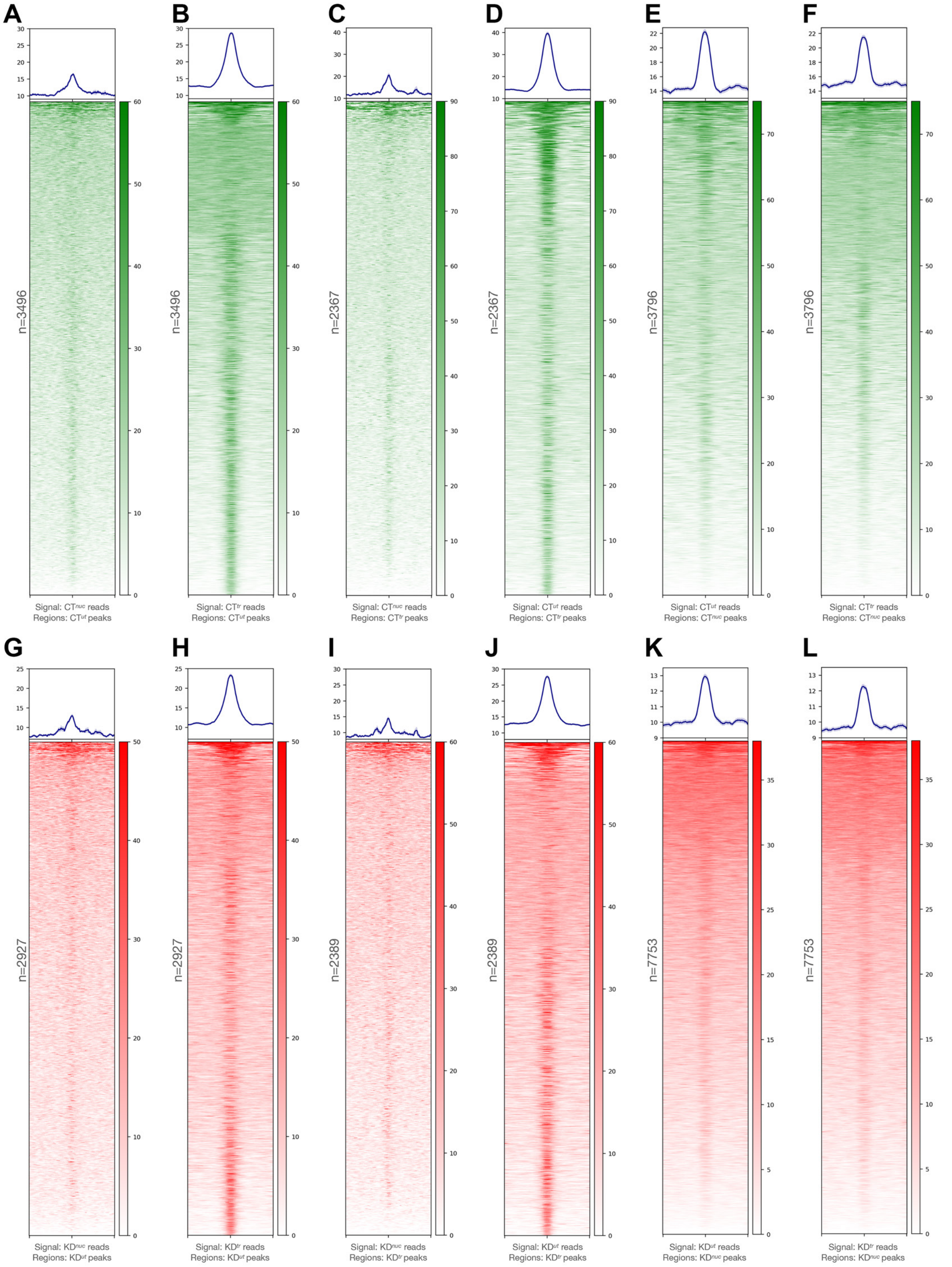

Supplementary Figure 17: A comparison of *AbC* G4-ChIP signals reciprocally on the peaks identified in CT and KD samples. (**A**–**F**) The *AbC* G4-ChIP signals were plotted reciprocally on peaks of CT samples and in the same order in the KD samples (**G**–**L**). The signals were plotted on the 5 kb flanks from centers of the peaks. The peaks identified in each sample of CT and KD show that other replicates also contain signals but weakly. Numbers indicate the peaks identified in the respective sample and the Y-axes of the plots have signals plotted as mean ± SEM.

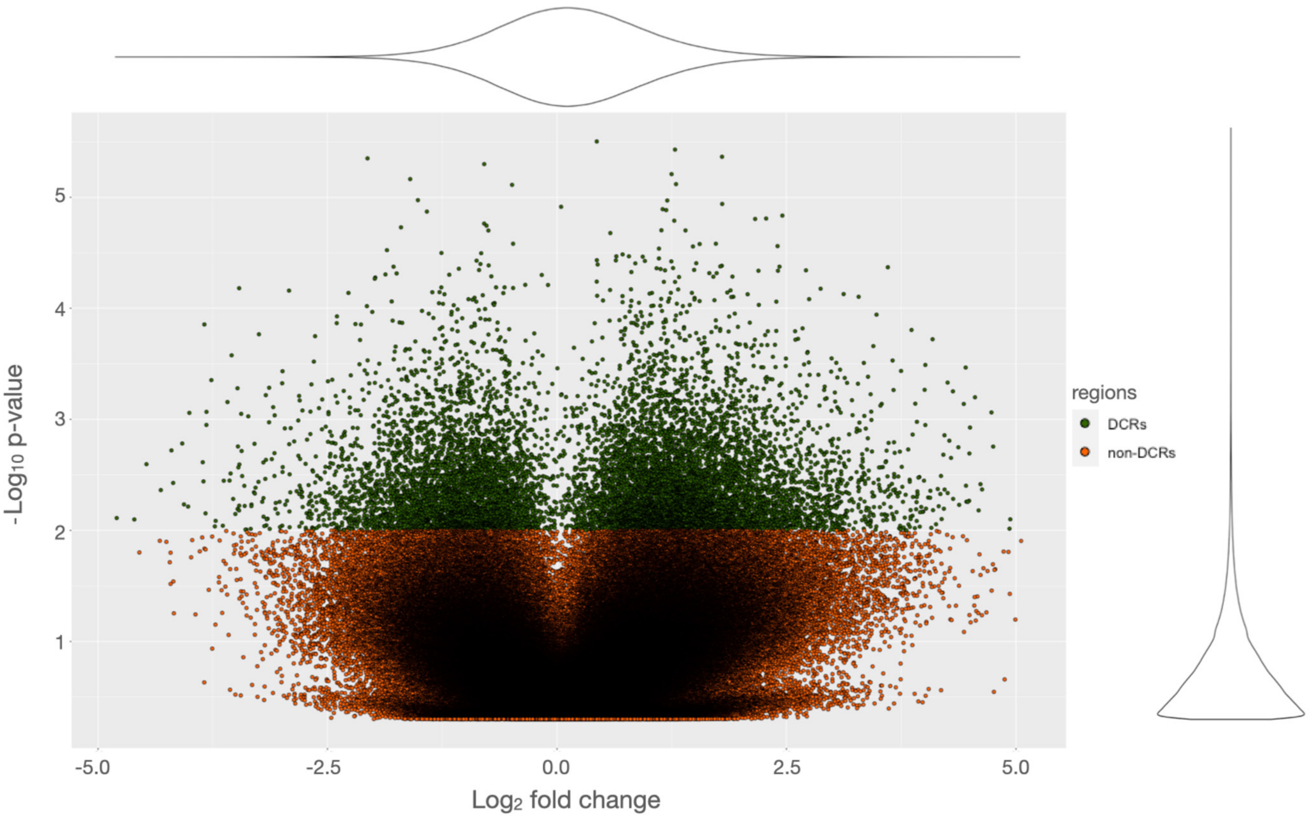

Supplementary Figure 18: A volcano plot of 0.2 kb genomic bins compared pairwise in triplicates between CT and KD. The X-axis represents $\log_2$ fold change calculated as KD/CT, and the corresponding *p-value*s on a $-\log_{10}$ scale are plotted on the Y-axis. The violin plots represent the density of the data points along the two axes. The red-green partition marks the non-DCRs and DCRs, respectively. The green data points correspond to the orange data points in Figure 4A.

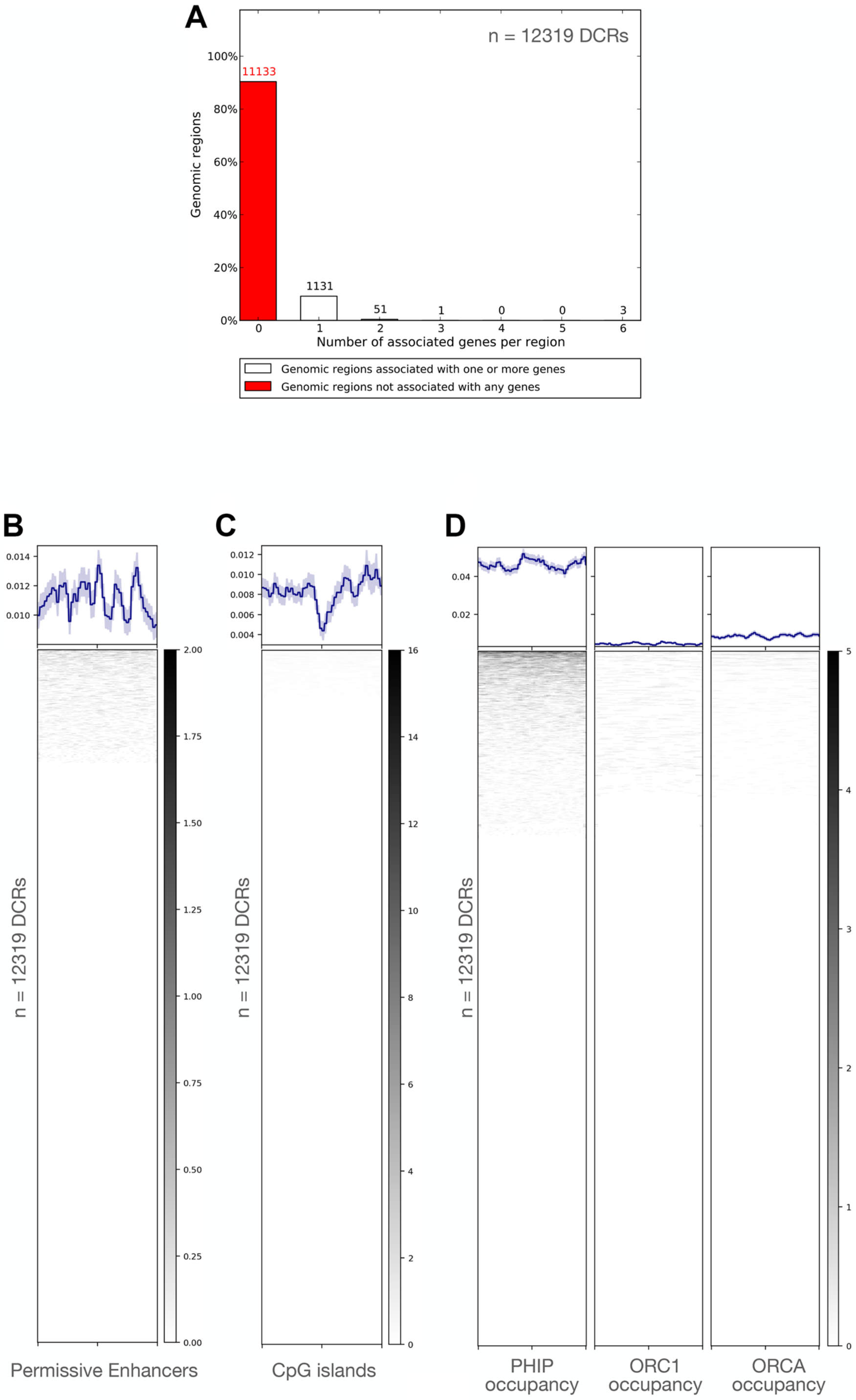

Supplementary Figure 19: Functional analysis of DCRs. (**A**) By using GREAT (web-based tool http://great.stanford.edu/) with an association condition of −5 kb to +1 kb from TSS for *cis* association and 10 kb in any direction for *trans* association, we found that nearly 90% of the DCRs are not associated with genes (red bar). (**B**, **C**) By plotting the occurrence of known permissive enhancers (FANTOM5) against the bed coordinates of DCRs, we found that the DCRs have no association with enhancers, with about 75% of DCRs having no known enhancers in 5 kb flanks (B). A similar analysis of the occurrence of CpG islands against the bed coordinates of DCRs showed that the DCRs are preferentially located in CpG island-poor regions. (**D**) The DCRs are also distant from the known origin of replication sites annotated by the mentioned markers (GSE28911, GSE37583 and GSE81165). Numbers indicate the regions identified in the respective sample, and the Y-axes of the plots have signals plotted as mean ± SEM.

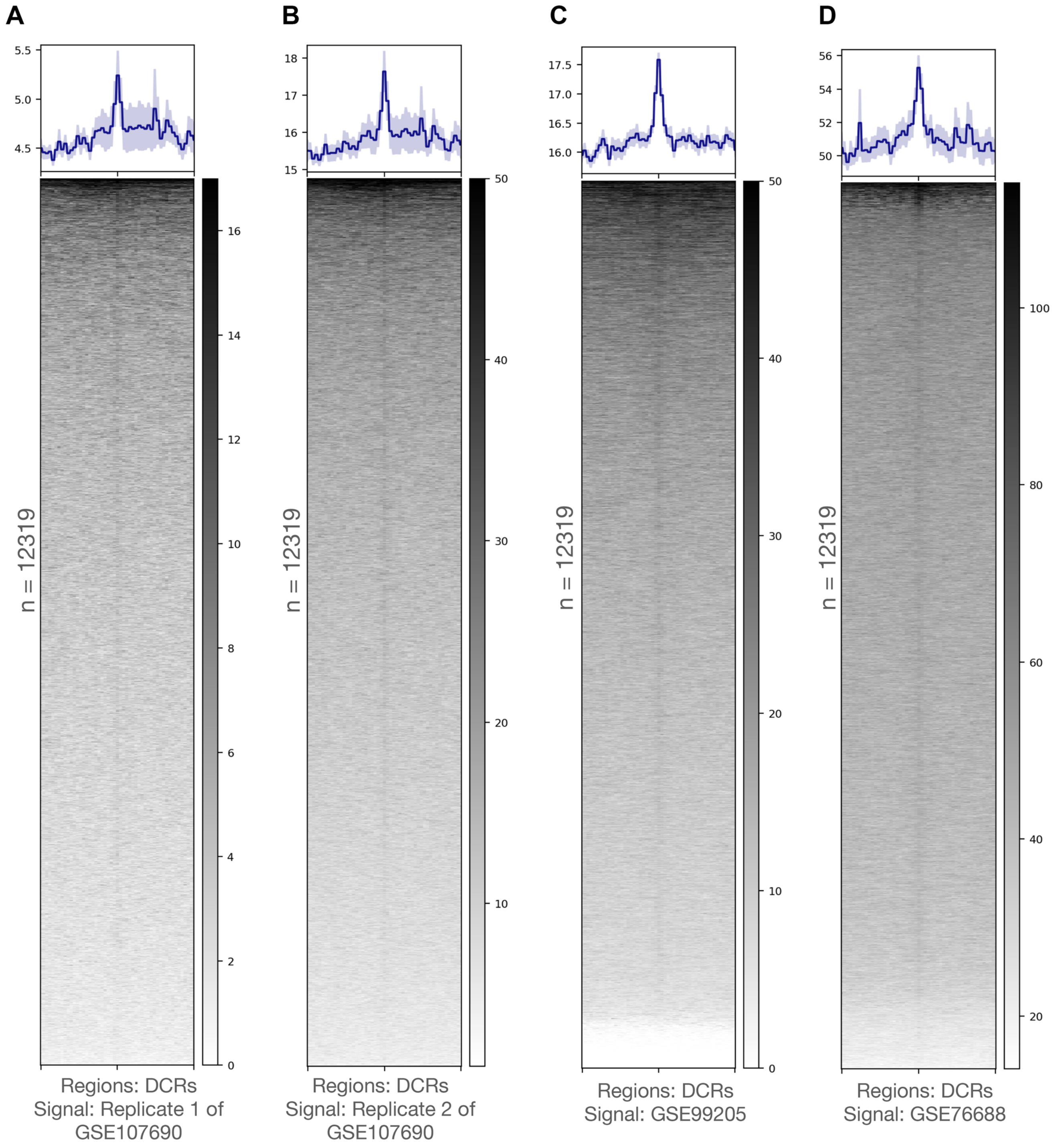

Supplementary Figure 20: Discovery of signals from publicly available G4-ChIP datasets. (**A**–**D**) The heat maps show the weak enrichment of G4 signals from independent experiments on the 5 kb flanks of the DCRs. This suggests that the DCRs identified from the current study are genuine G4-forming regions. Numbers indicate the regions identified in the respective sample, and the Y-axes of the plots have signals plotted as mean ± SEM. The signals have been derived from the GSE datasets mentioned below the heatmaps.

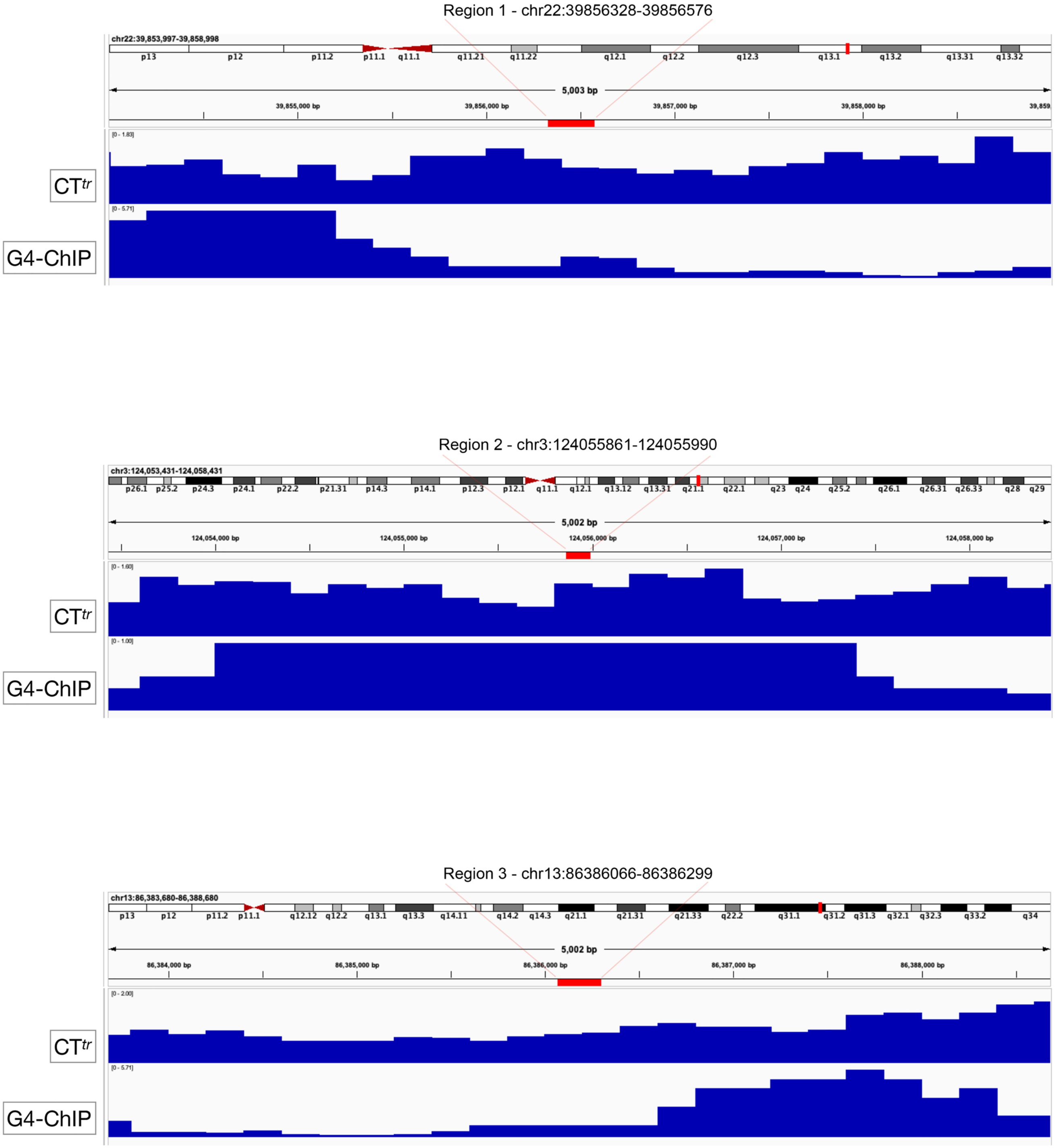

Supplementary Figure 21: IGV views of three candidate genomic regions identified as DCRs as well as pulled down in G4-ChIP. The signals are plotted in blue in a bin size of 0.2 kb. The Y-axes show pull-down to input ratios after normalizations for sequencing depth variation such that the larger samples were downsized to the depth of smaller samples using the bamCompare function in deepTools. The red bar indicates the genomic location of the region which can be amplified using PCR to establish the pull-down of these regions in multiple independent assays (not shown). The mean length of the sonicated chromatin used for immunoprecipitation is such that the indicated amplicon region will significantly overlap with the neighboring peaks of signals in both the samples. The names of the samples are indicated on the left. The Y-axis values are auto-scaled by IGV.

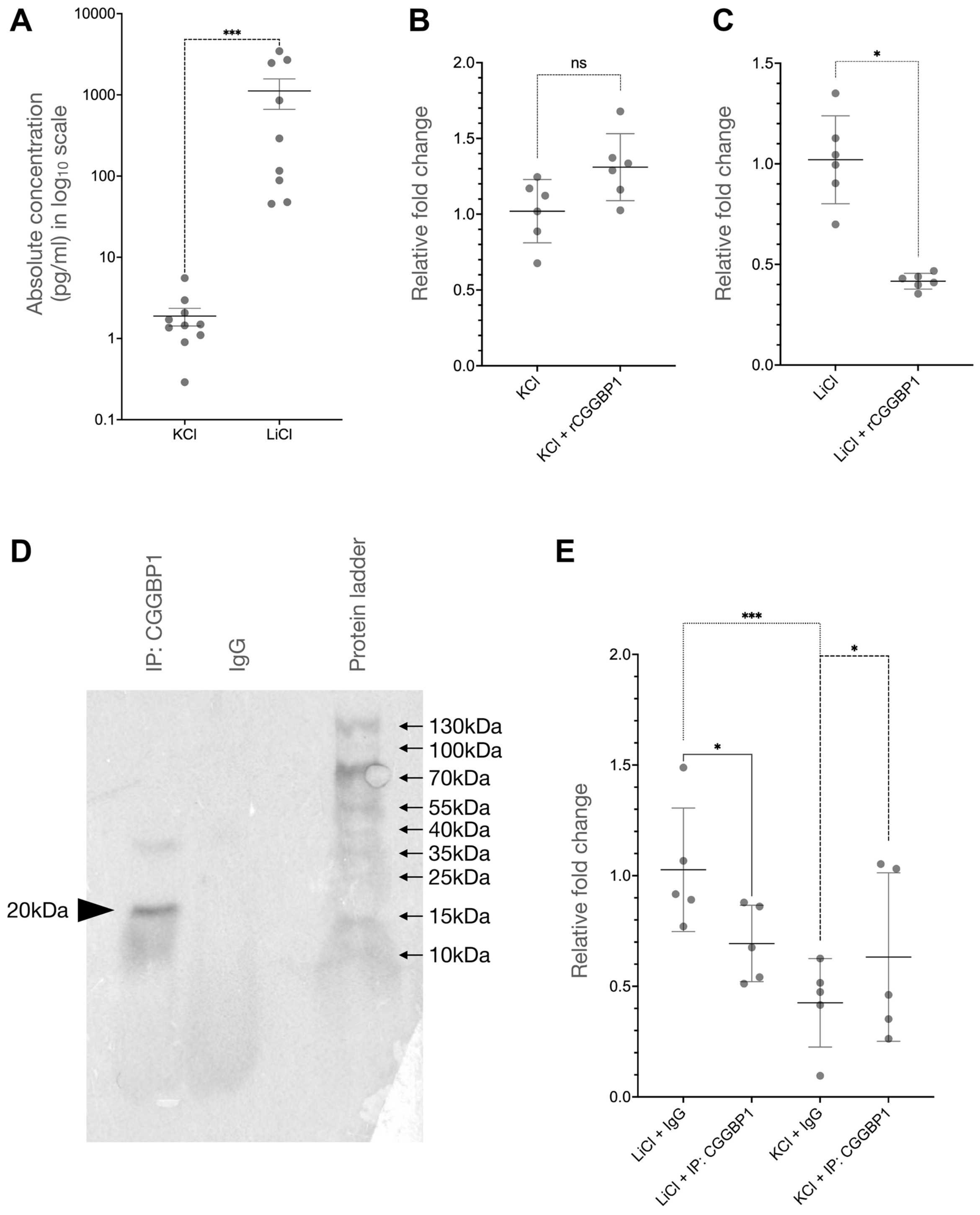

Supplementary Figure 22: Quantitative analysis of G4 formation on the Control DNA. (**A**) The Control DNA formed stable G4s in the presence of KCl and thus gave poor amplification in the polymerase stop assay compared to the sample of the Control DNA in the presence of LiCl. (**B**, **C**) The Control DNA was pre-incubated with rCGGBP1 first and then either incubated in the presence of KCl (B) or LiCl (C). A stronger polymerase stop was observed in the presence of LiCl (C) than KCl (B), which suggests that rCGGBP1 occupies the Control DNA, perhaps in the absence of G4s. (**D**) Western blot assay of immunoprecipitation of CGGBP1 from HEK293T cells under low salt condition. (**E**) Pre-incubation of the Control DNA with immunoprecipitated CGGBP1 yielded a similar effect of polymerase stop. A non-specific isotype immunoprecipitate was used as a negative control. In the presence of LiCl, where G4-formation is less favored, the polymerase stop showed a stronger effect when pre-incubated with immunoprecipitated CGGBP1 compared to KCl, wherein immunoprecipitated CGGBP1 did not yield a significant polymerase stop effect. The shaded circles indicate the replicates for respective samples, while the asterisk represents an order of significance level.

Supplementary Table 1: Summary of sequencing data acquired for *in vitro* G4 DNA-IPs, G4-ChIP and the corresponding inputs

Samples	Total reads sequenced	Reads run through Porechop	Mean length of reads run through Porechop	% mapped reads run through Porechop
**G4 DNA-IP*^{nat}***	9972207	9966894	1095.6	62.26
**G4 DNA-IP*^{denat}***	13487569	13401504	667.29	54.40
**G4-ChIP**	7089256	7083791	1040.75	34.42
**G4 DNA-IP Input**	5845120	5809383	605.19	63.29
**G4-ChIP Input**	5570204	5558589	829.10	56.05

Supplementary Table 2: Properties of peaks called on aligned sequences in the samples *in vitro* G4 DNA-IPs, G4-ChIP and CT^{nuc}

Samples	Total peak count	Total peak length (bp)	Average peak length (bp)
**G4 DNA-IP^{nat}**	226	676935	2995.29
**G4 DNA-IP^{denat}**	15709	33399525	2126.14
**G4-ChIP**	1455	3715550	2553.64
**CT^{nuc}**	3796	5315403	1400.26

Supplementary Table 3: Summary of *allquads* output on the sequences of *in vitro* G4 DNA-IPs, G4-ChIP, CT^{nuc} and the corresponding inputs

Samples	Reads subjected to *allquads*	Reads with G4 signature sequence identified by *allquads*	% Reads with G4 signature sequence identified by *allquads*
**G4 DNA-IP^{nat}**	9966894	1214180	12.18
**G4 DNA-IP^{denat}**	13401504	820014	6.12
**G4-ChIP**	7083791	640838	9.05
**CT^{nuc}**	45882202	1197364	2.61
**G4 DNA-IP Input**	5809383	341810	5.88
**G4-ChIP Input**	5558589	339856	6.11
***AbC* G4-ChIP Input**	40114795	1020311	2.54

Supplementary Table 4: Summary of *allquads* output on peaks called in *in vitro* G4 DNA-IPs, G4-ChIP and CT^{nuc}

Samples	Unique peaks with G4	Peak length with G4 signature (bp)	% peaks having G4 signature	% of peak lengths containing G4 signature
**G4 DNA-IP^{nat}**	41	10135	18.14	1.50
**G4 DNA-IP^{denat}**	3598	139154	22.90	0.42
**G4-ChIP**	189	16203	12.99	0.44
**CT^{nuc}**	380	35422	10.01	0.67

Supplementary Table 5: Summary of *allquads* output on previously published data (GSE99205 and GSE76688)

Samples	Reads trimmed for sequencing adapters	Reads with G4 signature sequence identified by *allquads*	% reads with G4 signature sequence identified by *allquads*
**HaCaT_Replicate1**	35539225	992034	2.79
**HaCaT_Replicate2**	46171793	1408214	3.05
**HaCaT_Replicate3**	134148412	3684342	2.75
**HaCaT_Input**	55700688	680770	1.22
**NHEK_Replicate1**	274391548	4647856	1.69
**NHEK_Replicate2**	276477916	5251417	1.90
**NHEK_Input**	179603728	2663305	1.48

Supplementary Table 6: Summary of sequence data for CT*nuc* and the corresponding input

Samples	Total reads sequenced	Total quality filtered reads (≥100 bp)	Basecount of quality filtered reads (≥100 bp)	Average length of quality filtered reads (bp)	% of quality filtered reads aligned using bowtie2
**CT*nuc***	77630217	45882202	7530031562	164.12	89.35
**Input*nuc***	71364574	40114795	6565787917	163.67	86.71

Supplementary Table 7: Summary of sequencing data acquired for KD*nuc*

Samples	Total reads sequenced	Total quality filtered reads (≥100 bp)	Basecount of quality filtered reads (≥100 bp)	Average length of quality filtered reads (bp)	% of quality filtered reads aligned using bowtie2
**KD*nuc***	77577363	43113118	6807407492	157.9	80.54

Supplementary Table 8: Fisher's exact test on the number of control DNA sequences recovered from CT^{tr}, KD^{tr}, CT^{ut} and KD^{ut}

**CT^{tr}**: 6		**CT^{ut}**: 31	
**KD^{tr}**: 390		**KD^{ut}**: 35	
"*P* value and statistical significance"			
" Test"	"Fisher's exact test"		
" *P* value"	<0.0001		
" *P* value summary"	****		
" One- or two-sided"	Two-sided		
" Statistically significant ($P < 0.05$)?"	Yes		
"Data analyzed"	TR	UT	Total
"CT"	6	31	37
"KD"	390	35	425
"Total"	396	66	462
"Percentage of row total"	TR	UT	
"CT"	16.22%	83.78%	
"KD"	91.76%	8.24%	
"Percentage of column total"	TR	UT	
"CT"	1.52%	46.97%	
"KD"	98.48%	53.03%	
"Percentage of grand total"	TR	UT	
"CT"	1.30%	6.71%	
"KD"	84.42%	7.58%	

Supplementary Table 9: Separately submitted as a spreadsheet. See Supplementary Table 9

Supplementary Table 10: SINE and LINE contents of 12319 DCRs (lowest *p*-values), non-DCRs (highest *p*-values) and randomly drawn 0.2kb genomic regions derived using RepeatMasker

Repeat types	DCRs	Non-DCRs	Random genomic regions
**Total SINEs**	5.86	10.66	12.01
**ALUs**	3.22	7.92	9.78
**MIRs**	2.61	2.71	2.2
**Total LINEs**	13.65	16.68	20.01
**L1**	11.05	13.03	16.98
**L2**	2.1	3.08	2.59
**L3**	0.29	0.39	0.3

Supplementary Table 11: DNA oligonucleotides used for the synthesis of the Control DNA and the carrier DNA

**Control DNA**	
Fragment_1	5′-TAATACGACTCACTATAGGGCACCCCTACATCGCAGCGGTCTTTC GGGCTAACGGGATCATGGGACTCAGG-3′
Fragment_2	5′-AACGGGATCATGGGACTCAGGGGACAGCCTCAAGCAACATGCCC AGTCCTGACCTTCAATAAGGAAGCAAA-3′
Fragment_3	5′-ACCTTCAATAAGGAAGCAAACTGGGAAGGAGGGTGTCAGGGATA AAGGGGAGTCCTAGTCAAGGTGTCGG-3′
Fragment_4	5′-AGTCCTAGTCAAGGTGTCGGATGTCCTAAGACTTATGATCATTTTC TTAGGGTCTAAGGGCTCGAGGGTC-3′
Fragment_5	5′-GGTCTAAGGGCTCGAGGGTCTGCGGGTCGGTTTCCTTCTAGAATT AGTATCTTCTATAGTGTCACCTAAAT-3′
Fragment_5_revComp	5′ATTTAGGTGACACTATAGAAGATACTAATTCTAGAAGGAAACCGAC CCGCAGACCCTCGAGCCCTTAGACC-3′
**Carrier DNA**	
Fragment_A	5′-TAATACGACTCACTATAGGGCACCCCTACATCGCAGCGGTCTTTC ATGCTAACATGATCATATGACTCAAT-3′
Fragment_B	5′-AACATGATCATATGACTCAATGGACAGCCTCAAGCAACATGCCCA GTCCTGACCTTCAATAAGGAAGCAAA-3′
Fragment_C	5′-ACCTTCAATAAGGAAGCAAACTATGAAGGAATGTGTCAATGATAA AATGGAGTCCTAGTCAAGGTGTCGG-3′
Fragment_D	5′-AGTCCTAGTCAAGGTGTCGGATGTCCTAAGACTTATGATCATTTT CTTAATGTCTAAATGCTCGAATGTC-3′
Fragment_E	5′-TGTCTAAATGCTCGAATGTCTGCATGTCGGTTTCCTTCTAGAATT AGTATCTTCTATAGTGTCACCTAAAT-3′
Fragment_E_revComp	5′-ATTTAGGTGACACTATAGAAGATACTAATTCTAGAAGGAAACCGA CATGCAGACATTCGAGCATTTAGACA-3′
